# Sleep Stage Classification in Children Using Self-Attention and Gaussian Noise Data Augmentation

**DOI:** 10.3390/s23073446

**Published:** 2023-03-25

**Authors:** Xinyu Huang, Kimiaki Shirahama, Muhammad Tausif Irshad, Muhammad Adeel Nisar, Artur Piet, Marcin Grzegorzek

**Affiliations:** 1 Institute of Medical Informatics, University of Lübeck, Ratzeburger Allee 160, 23562 Lübeck, Germany; 2 Department of Informatics, Kindai University, 3-4-1 Kowakae, Higashiosaka City 577-8502, Osaka, Japan; 3 Department of IT, University of the Punjab, Lahore 54000, Pakistan; 4 Department of Knowledge Engineering, University of Economics, Bogucicka 3, 40287 Katowice, Poland

**Keywords:** sleep stage classification in children, Gaussian noise data augmentation, data imbalance problem, self-attention mechanism, biomedical multivariate signal processing

## Abstract

The analysis of sleep stages for children plays an important role in early diagnosis and treatment. This paper introduces our sleep stage classification method addressing the following two challenges: the first is the data imbalance problem, i.e., the highly skewed class distribution with underrepresented minority classes. For this, a Gaussian Noise Data Augmentation (GNDA) algorithm was applied to polysomnography recordings to seek the balance of data sizes for different sleep stages. The second challenge is the difficulty in identifying a minority class of sleep stages, given their short sleep duration and similarities to other stages in terms of EEG characteristics. To overcome this, we developed a DeConvolution- and Self-Attention-based Model (DCSAM) which can inverse the feature map of a hidden layer to the input space to extract local features and extract the correlations between all possible pairs of features to distinguish sleep stages. The results on our dataset show that DCSAM based on GNDA obtains an accuracy of 90.26% and a macro F1-score of 86.51% which are higher than those of our previous method. We also tested DCSAM on a well-known public dataset—Sleep-EDFX—to prove whether it is applicable to sleep data from adults. It achieves a comparable performance to state-of-the-art methods, especially accuracies of 91.77%, 92.54%, 94.73%, and 95.30% for six-stage, five-stage, four-stage, and three-stage classification, respectively. These results imply that our DCSAM based on GNDA has a great potential to offer performance improvements in various medical domains by considering the data imbalance problems and correlations among features in time series data.

## 1. Introduction

Unlike in adults, sleep disorders in children are triggered by different internal and external predisposing factors at different ages and exhibit different phenotypic symptoms such as sleep terrors, obstructive sleep apnea, somnambulism, etc., and negative consequences such as irritability, behavioral problems, learning difficulties, etc. The length and depth of sleep and the duration of a sleep disorder also vary in different age groups. Difficulties in initiating and maintaining sleep are among the most common sleep problems in childhood. According to the study of [1], 41% of children aged 2 to 14 years in the clinical sample are assessed as having insomnia, and 31% of children aged six to thirteen years are reported to have disorders of initiating and maintaining sleep. Obstructive sleep apnea is prevalent among 5.8% of children with its onset being between two and eight years of age [2]. Obstructive sleep apnea is often accompanied by unusual sleep positions, sleep-related paradoxical breathing, night-time enuresis or diaphoresis, and morning headaches. Therefore, the study of sleep stage classification for child patients has far-reaching significance for improving the actual sleep conditions of infants and children.

Sleep stage classification is conducted by first recording *polysomnography* (PSG) signals, which consists of *electroencephalograms* (EEGs), *electrooculograms* (EOGs), *electromyograms* (EMGs), *electrocardiography* (ECG), etc. Subsequently, sleep experts analyze these multi-channel PSG recordings throughout the night and assess sleep stages every 30 s according to the *Rechtschaffen* and *Kales* (R & K) [3] and/or *American Academy of Sleep Medicine* (AASM) [4] criteria (the differences between the R & K and AASM standards and their electrode placements in EEG can be seen in our previous study [5]). R & K rules aptly categorize PSG recordings into seven stages, i.e., *Wakefulness* (WA), *Rapid Eye Movement* (R), *Stage 1* (S1), *Stage 2* (S2), *Stage 3* (S3), *Stage 4* (S4), and *Movement Time* (MT). Following AASM rules, PSG recordings are classified into five sleep stages, which include *Wakefulness* (W), *Rapid Eye Movement* (REM), *Non-REM1* (N1: sleep transition stage), *Non-REM2* (N2: light sleep), and *Non-REM3* (N3: deep sleep). However, manual scoring is constrained by the expertise of sleep experts and the monitoring instruments. It is vulnerable to human error, often time-consuming, and laborious. The agreement rate between expert consensus in sleep stage scoring was reported to be only about 90% [6], and when experts assessed PSG recordings a few weeks later, this agreement rate dropped to 75% [7], indicating the phenomenon of intra-expert variability. Accordingly, there is an urgent need to develop an automated sleep stage classification for children.

**Figure 1:**
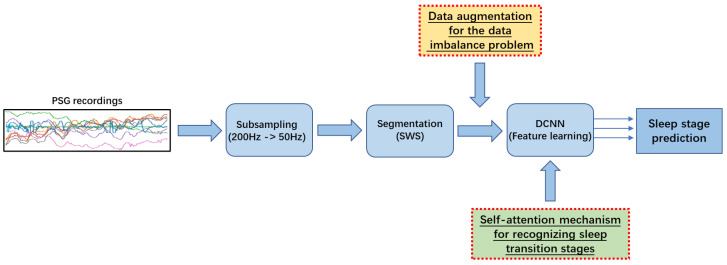
Schematic diagram of the optimized pattern recognition chain.

Figure 1 illustrates an overview of our previous study on sleep stage classification [5] (as shown in blue in Figure 1). In the first step of sleep stage classification, we took care of the sampling frequency because the original sampling frequency of the raw data is too high (200 Hz), Therefore, subsampling was used to reduce the redundancy in the input while obtaining enough valid information for sleep stage classification. Then, a *Sliding Window Segmentation* (SWS) [8] algorithm was used to collect segments of PSG recordings by sliding a time window of a fixed length with a certain stride. Subsequently, useful features were learned using a *DeConvolutional Neural Network* (DCNN), which includes an encoder based on a convolutional block and a decoder based on a deconvolutional block [9]. Finally, the DCNN predicted the sleep stage at each timestamp in the framework of multi-class classification. Despite the impressive experimental results in overall classification performance with DCNN, some limitations need to be further addressed. Without the extraction and analysis of representative and distinctive hand-crafted features, the DCNN cannot accurately detect the short-term sleep transition stage since it usually has a small proportion in the dataset and its sleep signal is constantly maintained at a low frequency, while there are no specific sleep waveforms. They are the main triggers for frequent misclassification between sleep stages.

Many studies have been implemented with relatively effective classification results, but still struggle with the problem caused by the sleep transition stage. A typical method for classifying sleep stages is the analysis of correlations between hand-crafted features for distinguishing sleep stages, such as the experiment performed by [10]. The correlation coefficient in the frequency domain was defined by *Empirical Mode Decomposition* (EMD) and the extraction and classification of EEG features were realized with *K*-means. Instead, Ref. [11] modified the experimental idea where the dimension-reduced EEG segments were transferred to the graph framework to analyze the correlation between the features and fed to an ensemble classifier to identify the sleep stages. In contrast, a *One-Dimension Convolutional Neural Network* (1D-CNN) was proposed by [12] for automatically learning multi-channel EEG and EOG features and their correlations. In [13], time-frequency features of EEG were extracted and the frequency evolution was learned using 3D-CNN and *Long-Short-Term Memory* (LSTM), respectively. There is also a study [14] in which transfer learning was used to attempt to transfer knowledge from a large dataset (source domain) to a small cohort (target domain) for automatic sleep stage classification, and accurate sleep classification based on a small number of subjects was achieved. Although the above experiments yielded good results in terms of overall accuracy, the problem of imbalance across sleep stages caused biased performance evaluations. Without applying the data-balanced strategy, these algorithms cannot effectively contribute to the detection of the sleep transition stage. In addition, the sleep characteristics of the sleep transition stage in EEG and EMG are also highly similar to those of the REM stage, which makes it difficult to identify sleep transition stages accurately.

Therefore, in accordance with the aforementioned shortcomings in existing studies, this paper addresses the following two issues in the sleep study: the first is the problem of data imbalance, i.e., the distribution of examples across classes is skewed. A typical classification assumes an equal number of examples for every class. A minority class consisting of a small number of examples provides fewer opportunities to capture its relationship to features. This leads to a model with poor classification performance for the minority class. For instance, we often find that a classifier achieves an accuracy close to 100% for majority classes, but it suffers a serious loss of accuracy for minority classes. Therefore, it is important to develop an approach for improving the classification accuracy of a minority class without seriously compromising the accuracy of a majority class. However, it is not always possible to collect more data. Therefore, an alternative solution to this problem should be sought. In our sleep stage classification, both N1 and REM are in a state of muscle relaxation and low-frequency brain waves [15]. Hence, it is difficult to visually distinguish between N1 and REM based on EEG and EMG. As a result, much smaller data can be collected for the N1 stage as compared to the other stages, thus leading to a data imbalance problem.

We adopt a data augmentation approach that simulates oversampling where the number of examples in a minority class is increased by creating modified copies of existing examples [16] (as shown in the yellow in Figure 1). One main data augmentation approach is noise insertion which enables a classification model to reduce overfitting by avoiding focusing on only a few features and extracting generalized combinations of features. In other words, the impact of noise insertion is similar to the one of weight regularization in training a classification model. Specifically, considering that *Gaussian Noise* (GN) is statistical noise in the normal distribution [17] and Gaussian random events are very common in nature, we implement *Gaussian Noise Data Augmentation* (GNDA) that injects GN into PSG recordings of each segment for a minority class during training [18]. GN with a mean of zero has data points essentially in all frequencies, effectively distorting high-frequency features. This also means that lower frequency components (expected data) will also be distorted, but neural networks can learn to outperform this, i.e., they use enough data to correctly learn statistically useful features for recognizing minority classes. This way, adding the right amount of noise can overcome the data imbalance problem.

**Figure 2:**
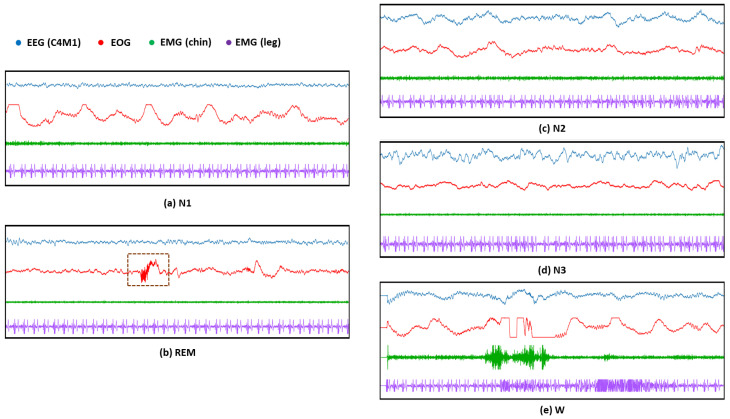
Examples of sleep patterns based on EEG, EOG, chin-EMG, and leg-EMG sensor modalities in 5 sleep stages on the SDCP dataset: (**a**) The instance of sleep patterns based on 4 sensor modalities in the N1 stage; (**b**) The instance of sleep patterns in the REM stage based on 4 sensor modalities, where the brown dashed box indicates the difference in sleep patterns that exist in the EOG modality of the REM stage compared to the other sleep stages; (**c**)The instance of sleep patterns based on 4 sensor modalities in the N2 stage; (**d**) The instance of sleep patterns based on 4 sensor modalities in the N3 stage; (**e**) The instance of sleep patterns based on 4 sensor modalities in the W stage.

The second issue is the difficulty of recognizing the N1 stage because it switches to other stages (e.g., W or N2 stages) in a very short period of time (such as 3 to 5 min). Moreover, as illustrated in Figure 2a,b, the EEG and even EMG features of the N1 stage are quite similar to those of the REM stage because only *Low Amplitude Mixed Frequency* (LAMF) brain waves occur in these two sleep stages without accompanying sleep features such as *K*-complexes and sleep spindle, and the entire body musculature is in a relaxed state. Therefore, although many existing methods only use EEG, it is difficult to accurately identify N1 and REM stages without forcibly extracting hand-crafted features. In general, it is necessary to use the sleep features in the EOG (as shown in the brown dashed box in Figure 2b) to further assist in distinguishing between these two sleep stages. As illustrated in the green in Figure 1, we employ thus a self-attention mechanism [19] that embeds the position information of the features and examines all possible pairs of features to extract useful local and global feature correlations. Specifically, the *DeConvolution- and Self-Attention-based Model* (DCSAM) is devised by incorporating the self-attention mechanism into a DCNN. The feature map obtained by the DCNN can be embedded with its position information. Then, the self-attention mechanism is used to perform fine-grained relativity analysis, which can evaluate the correlations between temporal features in the feature map to maximize the distinction between different sleep stages, so that the detection accuracy of the N1 stage is greatly improved.

Attention is a mechanism for simulating cognitive concentration. The idea is that the model focuses on important features while downplaying others. On this basis, the mechanism of self-attention not only provides performance improvement but also can serve as a tool for interpreting the behavior of models. The weights computed by the self-attention mechanism could inform us about important features in context. The experimental results on our own SDCP dataset [5] show that higher performances are accomplished when a minority class such as N1 is expanded using GNDA. Compared to DCNN and traditional RNN-based attention [20], our GNDA-based DCSAM can achieve impressive performance due to its superior ability to emphasize key features of each sleep stage by determining attention weights of all possible feature pairs. In addition, we demonstrate that GNDA-based DCSAM attains a comparative performance to state-of-the-art methods on the public Sleep-EDFX dataset [21,22]. This is further evidence of the generality and practicality of the GNDA-based DCSAM.

This paper is organized as follows: Section 2 shows the related studies tackling the problem of attention mechanism and goes through the existing studies to show the advantages of our GNDA-based DCSAM. Its methodological details are introduced in Section 3. Then, Section 4 presents the experimental datasets (SDCP and Sleep-EDFX), data processing, experimental settings, performance evaluation, and discussion. Finally, Section 5 outlines the conclusion and future improvements of our study. Apart from the main body of this paper, Appendices detail the Gaussian noise injection test, subsampling frequency test, and sensor channel test.

## 2. Related Work

The attention mechanism is one of the biggest recent advancements in neural network modeling. Just as the neural network attempts to mimic the actions of the human brain in a simplified way. The attention mechanism is also an attempt to implement the same action of selectively focusing on the relevant target areas while suppressing other useless information in deep neural networks. There are several attention mechanisms that are used in practice. Two conventionally used mechanisms are additive attention [23] and dot-product attention [19]. Additive attention is a form of attention mechanism that uses a fully connected network with a hidden layer to compute the attention weight of each feature. In comparison, dot-product attention computes the attention weight of each feature by the matrix product of input features. Indeed, additive and dot-product attentions have the same computational complexity, but the dot-product attention operation can use highly optimized parallel matrix multiplication. In addition, it can avoid the problem of a long-term dependence on RNN and significantly increase the parallel processing capability. The self-attention mechanism [19], also called intra-attention and is a variant of the attention model that uses the scaled dot-product to compute the attention weights. It has been widely applied in various fields, such as *Natural language processing* (NLP) [24], *Computer Vision* (CV) [25,26], and *Time Series Analysis* (TSA) [27,28]. Covering self-attention-based methods in various fields is out of the scope of this paper, and we focus only on those treating time series data.

Numerous time series models rely on simple regressive algorithms in place of deep learning [29,30,31]. Some motivations for this are interpretability, constrained data size, and low training cost. Attention mechanisms offer a compelling argument, and the results can be applied to explain the reasons for the performance enhancements. In the healthcare field, ref. [32] proposed an interpretable bidirectional recurrent neural network-based model (HAN-ECG) for identifying *Atrial fibrillation* (Afi) from ECG recordings. While it is a hierarchical attention network that features three attention mechanisms to achieve multi-resolution analysis in ECG leading to Afi. In [33], a 1-D convolution- and self-attention-based framework called *Simply Attend and Diagnose* (SAnD) with single- and multi-task strategies for multivariate medical time-series data was introduced since self-attention can check correlations between all timestamp pairs. However, one of the major difficulties lies in the consideration of long time series. A masking technique was adopted to address this by hiding timestamps that were too far in the past, as well as applying high-density interpolation rather than adding layer normalization following the self-attention module. Meanwhile, in [34], a novel temporal attention-based encoder-decoder model was developed for multivariate time series. It consists of *Bidirectional Long Short-Term Memory* (Bi-LSTM) layers with a temporal attention module to extract long-term dependencies and correlations between multivariate temporal channels. To analyze multivariate financial time series data, ref. [35] proposed an LSTM and attention-based financial time series prediction framework (AT-LSTM) to predict stock prices. The input features of a financial time series are first assigned different weights by the attention mechanism in each time period, and then all the weighted features constitute a feature sequence used in the LSTM prediction.

In many studies [36,37,38,39,40,41,42,43,44,45,46,47,48,49,50,51], feature engineering algorithms such as *Fourier Transform* (FT), *Wavelet Transform* (WT), *Spectral Features Analysis* (SFA), and *Time-frequency Analysis* (TA), etc., were used to generate and extract hand-crafted features from PSG recordings. Then various machine learning methods (e.g., *Support Vector Machine* (SVM), *Decision Tree* (DT), *Adaptive Boosting* (Adaboost) and RF, etc.) were applied to predict the sleep stage. Recently deep learning methods using LSTM, CNN, DCNN, and other variants could achieve strong performances on sleep stage classification [5,12,52,53,54]. Nevertheless, they have a serious limitation, namely the lack of interpretability. Some researchers have used an attention mechanism to explain classification results by discovering the correlations between different features in a long-term context.

Specifically, in [55], an autoencoder with a selective attention unit was proposed to focus on relevant inputs. The feature representation was learned over a large set of predefined handmade features for sleep stage recognition in training. Ref. [56] presented a unified hybrid self-attention model (HybridAtt) to identify sleep stages by calculating the feature correlations in the channel and temporal levels based on 14-channel PSG recordings. The multi-view convolutional representation mechanism learns channel-specific and global view features from input features and then uses attention mechanisms to fuse the features of multiple views by inferring their dependencies. In [57], they demonstrated a model that uses adversarial training along with attention mechanisms to extract transferable information in the cross-dataset scenario from two different EEG databases, namely *Physionet 2018 Challenge* (P18C) and *Sleep Heart Health Study* (SHHS). Casal et al. [58] proposed a two-layer architecture formed by a temporal CNN and a transformer to learn the transition rules between awake and asleep stages using only HR signals from a pulse oximeter. All of the aforementioned experiments verified the practicability of attention mechanisms for sleep stage analysis.

To our knowledge, the most similar study to ours is [54], which uses a single EEG channel (*Fpz-Cz*) for adult patients in the Sleep-EDF(X) database by analyzing non-overlapping segments to automatically identify sleep stages with a convolution- and sequential attention-based algorithm. However, the weights of each sleep stage are predefined, which limits the practical applicability of this algorithm in terms of scope and scenario and the experimental subjects are adults. There are only a small number of existing studies targeting sleep stage classification in children, and examples of them are as follows: The approach presented by [59] used a two-stacked SVM-based sleep-wake stage classification approach to distinguish Non-REM from REM and wakefulness from sleep based on the analysis of six standard time-frequency domain features of heart rate variability extracted from the *Photoplethysmogram* (PPG). The performance was not sufficient for clinical use. Ref. [60] collected data on facial expressions using a video during children’s sleep. Behavioral changes in the facial region were used for sleep-wake states by using a CNN to extract the trainable features and employing SVM to classify the sleep stages. In comparison with this video-based sleep stage detection and to highlight the advantages of non-contact data acquisition, a non-contact sensing technology, namely *Impulse-radio Ultrawideband* (IR-UWB) radar [61], was used to acquire fine movement asymmetries and vital signs in children. Subsequently, radar data were analyzed with a sleep-wake decision algorithm accompanied by amplitude-integrated EEG, respiratory signals, and movement features. Ref. [62] also performed an IR-UWB radar-based sleep stage classification in children undergoing PSG (e.g., EEG, ECG, thermistor, plethysmography, *Pulse Oximetry* (SpO2), etc.) and wakefulness and sleep states can be well detected by applying an adaptive boosting method. Nevertheless, it is evident that each of the aforementioned studies did not classify sleep stages in detail, especially lacking identification of the N1 stage, but rather merged sleep stages, conducted only a binary classification task (sleep and awake), a three-stage task (W, REM, and Non-REM) and a four-stage task (W, REM, light sleep, and deep sleep). Despite the improved performance, this simplified classification has led to a research gap for effective sleep recognition of the N1 stage in children. In contrast, in [63], an experiment for the classification of the N1 stage in children was performed. A multi-domain hybrid neural network (HNN-Multi) consisting of CNN and LSTM was developed to implement a three-stage (N1, N2, and W) classification task based on the EEG signals. An effective combination of temporal and spatial time-domain features with time-varying frequencies was exploited and a performance improvement was achieved. The N1 stage is indeed the sleep transition stage midway between W and N2 stages, but identification of the N1 stage under conditions that ignore the influence of the REM stage is strongly biased.

In comparison, our DCSAM approach focuses on children and utilizes the self-attention mechanism based on multiple channels of PSG recordings to extract local inter-and intra-epochal features and implemented the standardized five-stage classification. On the one hand, multiple channels are crucial for sleep stage classification due to their unique signal phenotypes such as sleep spindles, *K*-complexes, slow-wave, etc. These phenotypes, which play an auxiliary role, are not based only on EEG, EOG, and EMG. Therefore, it is better to use multiple channels to yield good results. On the other hand, we performed GNDA to balance the class proportion to overcome the imbalanced data problem and discover the correlation of key features in different sleep stages using a self-attention mechanism, which significantly improves the detection accuracy of the sleep transition stage (N1 stage), rather than extracting hand-crafted features to gain a better understanding of sleep stage classification.

## 3. Methodology

Our DCSAM consists of a DCNN (Please see our previous study [5] for further explanation and calculation details of DCNN) and self-attention mechanism. The convolution [64] transforms an input segment into a high-level feature map and the deconvolution [65] further expands them by recovering latent features that were ignored in the former block. The self-attention mechanism then computes and updates explainable key features of sleep stages by calculating the attention weights of local features with their global dependencies. The following description begins with the introduction of the single-head self-attention mechanism. Afterwards, the multi-head self-attention mechanisms are briefly explained. The architecture of our proposed DCSAM and its implementation details are presented in the last part of this section.

### DeConvolution- and Self-Attention-Based Model

**Figure 3:**
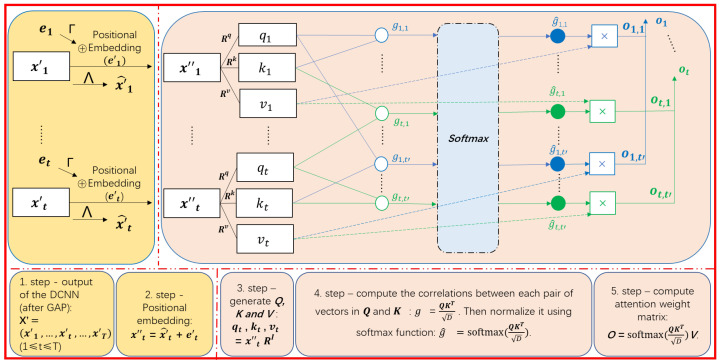
An illustration of the single-head self-attention mechanism: $X^{\prime }$ is the input matrix of dimensions T×C, where *T*, *C* represent the length of the time window and the number of channels, respectively. Γ and Λ are the transformation weight matrices used to convert the position vector E and the input vector $X^{\prime }$ after concatenation into a new input vector $X^{\prime \prime }$ encoded by the temporal position. Three weight matrices $R^{q}$, $R^{k}$ and $R^{v}$ used to generate corresponding *query* ($q_{t}$), *key* ($k_{t}$) and *value* ($v_{t}$) based on $X^{\prime \prime }$. *g* is the initial attention score that reflects the relevance between a given *query* and each *key* and is then normalized by softmax to produce $\hat{g}$. The attention weight o can be calculated by the sum of the multiplication between all $\hat{g}$s and its corresponding *values*.

Figure 3 portrays the computation of a single-head self-attention mechanism. The input and output of the self-attention mechanism are sequences. In particular, the generation of the output can be performed in parallel, since it is an advanced batch-processing algorithm that performs dot product operations between matrices. We assume that the output of the last deconvolutional layer of the DCNN is a feature map, which is defined as a sequence of matrices $X=x_{1},\cdots ,x_{T}$ where $x_{t}$ (1≤t≤T) is a C×M matrix created by vertically stacking an *M*-dimensional transposed vector $x_{t,c}$ (1≤c≤C) (i.e., the *c*th row of $x_{t}$ is $x_{t,c}^{T}$). Here, *T*, *C*, and *M* are the length of the feature map, the number of channels, and the number of filters of the last DCNN layer, respectively. *Global Average Pooling* (GAP) is performed to summarise $x_{t,c}$ into its average $x_{t,c}^{\prime }$. According to this, $x_{t}$ is converted into an *C*-dimensional vector ${x^{\prime }}_{t}={\left(x_{t,1}^{\prime },\cdots ,x_{t,C}^{\prime }\right)}^{T}$, and X is transformed into a matrix of dimensions T×C where it contains *T* vectors of *C*-dimensions such as $X^{\prime }$ = ${x^{\prime }}_{1},\cdots ,{x^{\prime }}_{T}$. This kind of GAP is useful for making our model more robust and resistant to overfitting while preserving the useful information for each channel. $X^{\prime }$ is fed as input to the self-attention mechanism to extract the correlations between all pairs of features in the local or global context.

As illustrated in Figure 3, the first step of self-attention is to perform position embedding [19] to take into account the temporal positions in $X^{\prime }$. Specifically, a *C*-dimension vector $e_{t}={\left(e_{t,1},\cdots ,e_{C}\right)}^{T}$ encoding temporal positions are defined by computing each dimension as follows:

(1) $$\begin{array}{c} \begin{array}{c} e_{t,2c^{\prime }}=\sin\left(\frac{t}{10,000^{2c^{\prime }/C}}\right)\;\;\;\mathrm{for}\;a\;\mathrm{dimension}\;\mathrm{with}\;\mathrm{an}\;\mathrm{even}\;\mathrm{number}\;\mathrm{index}\\ e_{t,2c^{\prime }+1}=\cos\left(\frac{t}{10,000^{2c^{\prime }/C}}\right)\;\;\;\mathrm{for}\;a\;\mathrm{dimension}\;\mathrm{with}\;\mathrm{an}\;\mathrm{odd}\;\mathrm{number}\;\mathrm{index}, \end{array} \end{array}$$

Unlike traditional recurrent neural networks, where each input is processed according to the order of timestamps, a self-attention mechanism shows similar attention weights for all inputs that have similar initial feature vectors when no position information is provided because all inputs are processed simultaneously. Therefore, position embedding is used to give the order context to the non-recurrent architecture. For our experiment, *t* represents the timestamp (absolute position) and the use of sine and cosine functions with different frequencies (temporal positions) make each timestamp characterized by a unique vector $e_{t}$ since the sine and cosine functions are stable in their periodicity and the embedding has a certain invariance and the wavelengths of sine and cosine range from 2π to 10,000·2π in different dimensions, which distinguishes the form of the functions in odd and even dimensions $2c^{\prime }$ and $2c^{\prime }+1$. The choice of $\frac{t}{{10,000}^{2c^{\prime }/C}}$ respects Equation (1) and ensures that *t*-second queries are possible even for long segments [19,66].

Then, $e_{t}$ is enhanced into a higher-level feature ${e^{\prime }}_{t}$ by multiplying weight matrix Γ with a C×C, that is, ${e^{\prime }}_{t}=\Gamma e_{t}$. In addition, ${x^{\prime }}_{t}$ is refined into a higher-level feature ${\hat{x^{\prime }}}_{t}$ by multiplying it with a C×C weight matrix Λ, that is, ${\hat{x^{\prime }}}_{t}=\Lambda {x^{\prime }}_{t}$. Then, $X^{\prime }$ is transformed into $X^{\prime \prime }$ = ${x^{\prime \prime }}_{1},\cdots ,{x^{\prime \prime }}_{T}$ where ${x^{\prime \prime }}_{t}={\hat{x^{\prime }}}_{t}+{e^{\prime }}_{t}$ encodes the characteristic of the *t*th temporal position.

As shown in Figure 3, the next step of the self-attention is to generate the *query* $q_{t}$, *key* $k_{t}$ and *value* $v_{t}$ by the following multiplication of ${x^{\prime \prime }}_{t}$ in $X^{\prime \prime }$ with three weight matrices $R^{q}$, $R^{k}$ and $R^{v}$, respectively.

(2) $$q_{t}=\;{x^{\prime \prime }}_{t}R^{q},$$

(3) $$k_{t}=\;{x^{\prime \prime }}_{t}R^{k},$$

(4) $$v_{t}=\;{x^{\prime \prime }}_{t}R^{v},$$

Here, there are two settings to define $R^{q}$, $R^{k}$, and $R^{v}$. The first is to consider $R^{q}=R^{k}=R^{v}$, and the second is to define them as different matrices. The second setting based on different projections has a higher expressiveness power than the first one, but the computational complexity of the former is much higher. Since the experimental performance is not significantly different between these settings, we decided to use the first one for the experiment. In addition, the multi-head self-attention mechanism described below performs multiple projections of ${x^{\prime \prime }}_{t}$ in a similar way as the second setting (although they are not exactly the same).

As depicted in the center of Figure 3, the correlation between ${x^{\prime \prime }}_{t}$ and ${x^{\prime \prime }}_{t^{\prime }}$ is quantified as the following attention weight ${\hat{g}}_{t,t^{\prime }}$:

(5) $$g_{t,t^{\prime }}=\;\frac{q_{t}\cdot {k_{t^{\prime }}}^{T}}{\sqrt{D}},$$

(6) $${\hat{g}}_{t,t^{\prime }}=\;\frac{\exp\left(g_{t,t^{\prime }}\right)}{\sum_{j}\exp\left(g_{t,j}\right)}\;\;\;\left(1\leq j\leq T\right),$$

where the initial attention $g_{t,t^{\prime }}$ is computed as the dot-product between $q_{t}$ and $k_{t^{\prime }}$ that indicates their similarity. Here, the dot-product tends to be unfavorably large as the increase of *D* that is the dimensionality of $q_{t}$ and $k_{t^{\prime }}$. So, the dot-product is scaled by $1/\sqrt{D}$. Afterward, as described in Equation (6), a softmax operation is employed to convert $g_{t,t^{\prime }}$ into ${\hat{g}}_{t,t^{\prime }}$ so that ${\hat{g}}_{t,1},\cdots ,{\hat{g}}_{t,T}$ are regarded as probabilistic values, each of which indicates the strength of the correlation of the feature ${x^{\prime \prime }}_{t}$ to the feature at another timestamp.

As represented by the projection of ${x^{\prime \prime }}_{t}$ by $R^{v}$ in Equation (4), $v_{t}$ is considered as a higher-level feature for ${x^{\prime \prime }}_{t}$. The dotted line in Figure 3 illustrates that a further higher-level feature $o_{t}$ for ${x^{\prime \prime }}_{t}$ is computed as the weighted mean of $v_{1},\cdots ,v_{T}$ using the corresponding attention weights ${\hat{g}}_{t,1},\cdots ,{\hat{g}}_{t,T}$, as formulated in the equation below:

(7) $$o_{t}=\;\sum_{t^{\prime }=1}^{T}\hat{g_{t,t^{\prime }}}v_{t^{\prime }},$$

Let us re-define $X^{\prime \prime }$ as a T×C matrix where the *t*th row is ${x^{\prime \prime }}_{t}^{T}$, and adopt a T×D matrix O where the *t*th row is $o_{t}^{T}$. The single-head self-attention mechanism to compute O from $X^{\prime \prime }$ in batch can be performed by the following matrix operations [19]:

(8) $$O=\;softmax\left(\frac{QK^{T}}{\sqrt{D}}\right)V,$$

where Q, K, V are T×D matrices that are created by vertically stacking $q_{t}$s, $k_{t}$s and $v_{t}$s for all timestamps. To summarize, the single-head attention takes a sequence of features $X^{\prime \prime }$ as input and outputs a sequence of higher-level features O by aggregating projected features at all timestamps V based on attention weights computed as $softmax(QK^{T}/\sqrt{D})$.

**Figure 4:**
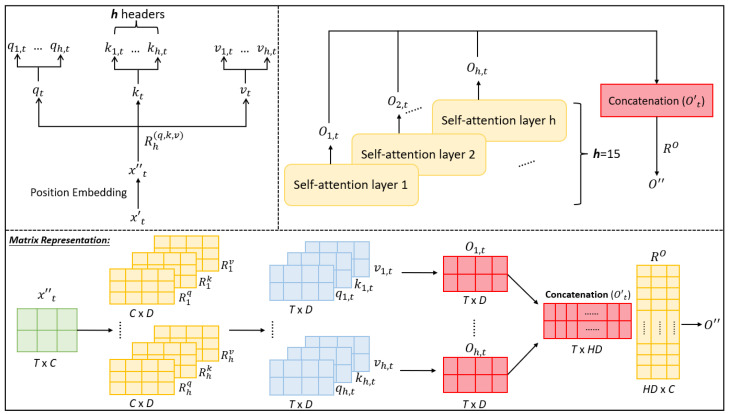
An illustration of the multi-head self-attention mechanism: input matrix $X^{\prime \prime }$ of dimension *T* X *C* and then converted into positional-encoded *query*, *key*, and *value* by multiplying corresponding $R^{\left(q,k,v\right)}$, which can be further decomposed into *h* sub-heads used to compute the final attention weight matrix $o^{\prime \prime }$ in terms of the mechanism of the single-head self-attention (see Figure 3), where each sub-head can focus on specific areas of attention.

It is easy to extend the single-head self-attention mechanism to the multi-head one. Specifically, the latter just executes the former *H* times using *H* sets of weight matrices ${\left\{(R_{h}^{q},R_{h}^{k},R_{h}^{v})\right\}}_{h=1}^{H}$. That is, different numbers of subheads $q_{h,t}$, $k_{h,t}$ and $v_{h,t}$ can be computed defer to the extended formulas $q_{h,t}={x^{\prime \prime }}_{t}R_{h}^{q}$, $k_{h,t}={x^{\prime \prime }}_{t}R_{h}^{k}$, and $v_{h,t}={x^{\prime \prime }}_{t}R_{h}^{v}$. As same as the single-head self-attention, all initial attention weights $g_{h,t}$ can be computed using scaled dot-product between each $q_{h,t}$ and the corresponding $k_{h,t}$ and its normalized attention weight ${\hat{g}}_{h,t}$ is produced by a softmax layer. Let $o_{h,t}$ be the higher-level feature for ${x^{\prime \prime }}_{t}$ with the T×D dimension generated by the *h*th single-head self-attention mechanism (i.e., *h*th head) (as shown in Equation (9)). The overall higher-level feature ${o^{\prime }}_{t}$ for ${x^{\prime \prime }}_{t}$ is obtained as an HD-dimensional vector created by concatenating $o_{1,t},\cdots ,o_{H,T}$ (as defined in Equation (10)). Last, a learnable weight matrix $R^{O}$ is multiplied with ${o^{\prime }}_{t}$ to produce a final output feature $o^{\prime \prime }$ that has the same dimensionality *C* to the input feature ${x^{\prime \prime }}_{t}$. The schematic diagram of the multi-head self-attention mechanism is depicted in Figure 4. Its advantage is that different heads could focus on different attention ranges so that the local and global correlations could be observed.

(9) $$o_{h,t}=\;softmax\left(\frac{q_{h,t}k_{h,t}^{T}}{\sqrt{D}}\right)v_{h,t}=\left[\begin{array}{c} o_{h,1}^{T}\\ \vdots \\ o_{h,t}^{T}\\ \vdots \\ o_{h,T}^{T} \end{array}\right],$$

(10) $${o^{\prime }}_{t}=\left[\begin{array}{c} \left[\begin{array}{c} o_{1,1}^{T}\\ \vdots \\ o_{1,t}^{T}\\ \vdots \\ o_{1,T}^{T} \end{array}\right]\cdots \left[\begin{array}{c} o_{h,1}^{T}\\ \vdots \\ o_{h,t}^{T}\\ \vdots \\ o_{h,T}^{T} \end{array}\right]\cdots \left[\begin{array}{c} o_{H,1}^{T}\\ \vdots \\ o_{H,t}^{T}\\ \vdots \\ o_{H,T}^{T} \end{array}\right] \end{array}\right].$$

**Figure 5:**
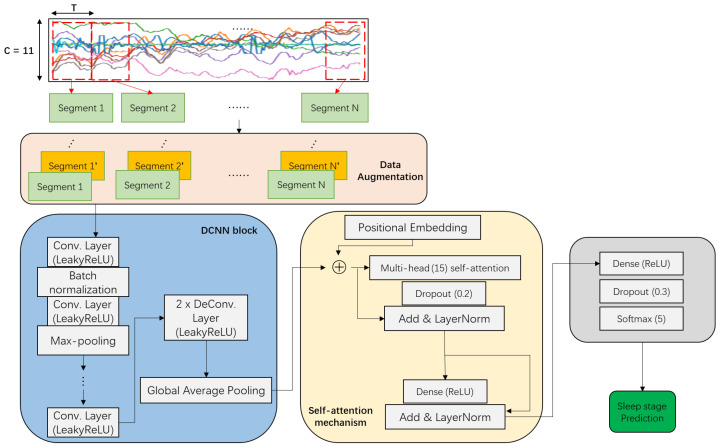
An overview of our proposed DCSAM for sleep stage classification.

Our DCSAM is illustrated in Figure 5 and its implementation detail is shown in Table 1. The input of the first convolutional layer is a tensor feature map of shape T×C, where *T* represents the length of the sliding window and *C* represents the number of sensor channels. A *Leaky Rectified Linear Units* (LeakyReLU) activation function is used in each convolutional and deconvolutional layer. It is a variant of normal ReLU activation and does not reach its saturation state as easily and avoids gradient dispersion. Batch normalization is applied immediately after the first convolutional layer to normalize the layer’s inputs by re-centering and re-scaling them, thereby avoiding the problem of internal covariate shift, which causes hidden layers of the network to have to learn to adapt to the new distribution when the input distribution changes. As a result, converging to a global minimum during the training process is difficult. In addition, to avoid overfitting and to maintain invariance of translation and scaling, the Max-Pooling layers are used in the convolution block. A residual connection and layer normalization [67] are used to add the output of the previous attention mechanism to the input of this layer and the sum is normalized. Then, an additional fully-connected dense layer with a softmax activation function is employed to predict sleep stage labels at timestamp *t*. Moreover, the dropout layer is inserted between dense and softmax layers to prevent the occurrence of overfitting. Finally, categorical crossentropy on the softmax layer is used as a loss function.

## 4. Experiments

In this section, we first give an overview of two datasets used in our experiments, and then data preprocessing is presented. Next, we introduce an RNN-based attention mechanism as the baseline model used for comparison with our DCSAM model and also provide our experimental setups. We then compare the final performances based on *k*-fold cross-validation and leave-one-subject-out cross-validation between DWT wavelets-based SVM, DCNN, RNN-based attention mechanism, multi-head self-attention mechanism, and our DCSAM with and without using GNDA. To show the generalization capabilities of our GNDA-based DCSAM, a comparison experiment on the public Sleep-EDFX dataset is also performed. At the end of this section, a discussion of the obtained results is presented. In addition, the Gaussian noise injection test, subsampling frequency test, and the sensor channel test based on the SDCP dataset as complementary experiments are shown in the Appendix A, Appendix B and Appendix C, respectively.

### 4.1. SDCP Dataset

#### 4.1.1. Dataset Description

**Figure 6:**
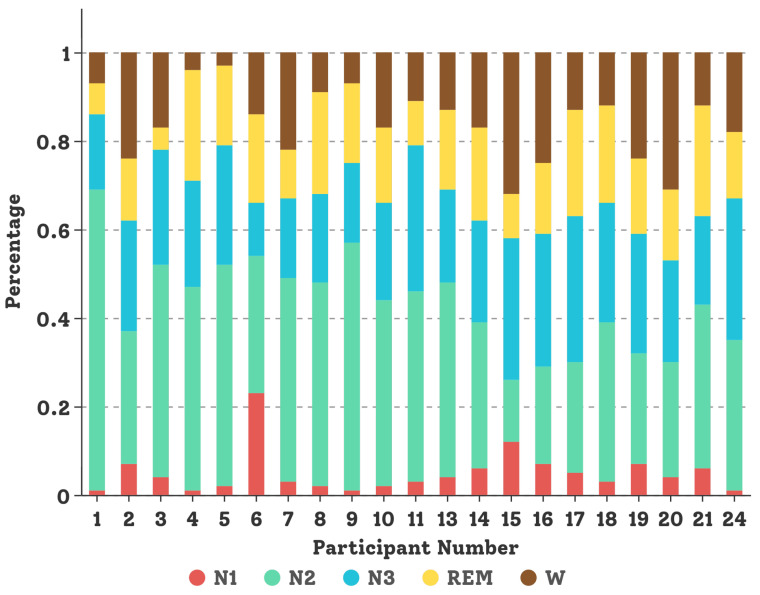
Data distribution for each child in the SDCP dataset.

The SDCP dataset [5] contains multi-channel PSG recordings for 21 subjects aged from 4 to 10 years old (14 females and 7 males) with sleep disorders. EEG and EOG channels were recorded using a sliver chloride sensor, namely *Ambu${}^{®}$ Neuroline Cup electrode* [68], and EMG channels were recorded from a hairless skin sensor sticker *Ambu${}^{®}$Neuroline-720* [68]. All sleep PSG recordings were stored in the *Philips Sleepware G3* [69] workstation and subsequently evaluated visually and manually by sleep experts. Demographic information about children and data distribution per child in the SDCP dataset are shown in Table 2 and Figure 6, respectively. The length of the multi-channel PSG recording is around 10 h from the evening to the next morning. For our experiment, four sensor modalities were selected, giving a total of 11 channels, e.g., 6 EEG channels (*O1M2, O2M1, F3M2, F4M1, C3M2, C4M1*), two EOG channels (left and right rapid eye movements), one chin EMG channel, and double legs EMG channels. The sampling frequency is 200 Hz and all recorded data were expertly labeled every 30 s to represent one of five sleep stages (e.g., W, REM, N1, N2, and N3) according to the AASM scoring standard. In the experiment, data collected from 17 subjects were used to train our DCSAM model, and data from the remaining four subjects (P1, P4, P9, P24) were used to test the performance and fine-tune the hyper-parameter of the model.

#### 4.1.2. Data Preprocessing

Since the original sampling frequency of our sleep data was 200 Hz, 10 and 11 multi-channel PSG recordings contained approximately 79,200,000 values. This is too large in terms of the computing power required to train our models. Relatedly, sleep stages do not change suddenly, so 200 values sampled in one second are redundant and may contain some unaltered superfluous information. Therefore, we subsampled the data by referring to distinct functional subband frequencies of EEG, EOG, and EMG (i.e., EEG data can be decomposed into functionally distinct frequency subbands, such as delta (0.5–3.9 Hz), theta (4–7.9 Hz), alpha (8–12 Hz) and beta (12–30 Hz), EOG (0.2–15 Hz) and EMG (15–45 Hz)). We tested three different subsampling frequencies (*SF*: 5 Hz, 10 Hz, and 50 Hz) in our experiments, and decided to use a sampling rate of 50 Hz.

After subsampling, GNDA described in Section 1 was applied to increase the proportion of the N1 stage and to avoid misclassification between the N1 stage and other sleep stages. A GN (Gaussian Noise), defined by a mean μ of zero and a standard deviation δ, can be generated. The δ controls the degree of dispersion of the GN and can be set according to the scale of each input *x*. A too-small δ has no effect, while a too-large δ makes the mapping function too difficult to learn. Various δs were used to fine-tune a pretrained DCNN in our previous study with GNDA in order to find the optimal δ. In particular, multiple δs were used to generate diverse training data that were useful for accurate classification. Based on the preliminary selection results shown in Appendix A, the δs are predetermined to (0.4), (0.2,0.4), and (0.2,0.4,0.6). Each δ was used to generate the same number of data as the original data of the N1 stage, so its proportion was expanded by a factor of two, three, or four as more δs were used. As provided in Table 3, after applying GNDA, the proportion of the N1 stage in the training set was close to that of the REM stage. Since the data in different channels have different ranges of values, we normalized the data in each channel to have zero mean and unit variance. This enabled us to tune δ regardless of data ranges in the different channels.

The following two settings of the SWS (Sliding Window Segmentation) process were used based on our preliminary experiments. In the first setting, the length *T* and the sliding stride ΔS of a time window were set to 30 and 30 s, respectively. This resulted in dividing PSG recordings into non-overlapping segments. The second setting was defined by T=300 and ΔS=30 to generate overlapping feature segments.

### 4.2. Sleep-EDFX Dataset

Sleep-EDFX [21,22] is a well-known public database that contains 197 whole-night sleep PSG recordings, including EEG (*Pz-Oz* and *Fpz-Cz*) and horizontal EOG. In *Sleep Cassette Study* (SC), 153 recordings were recorded between 1987 and 1991 to study the effects of age on the sleep of 25 to 101-years old healthy Caucasians and, in the *Sleep Telemetry Study* (ST), 44 recordings were collected in 1994 to research the effects of temazepam medications on the sleep of 22 healthy Caucasians with mild difficulties falling asleep. Each subject in the SC study applied a total of about 20 h of PSG sleep recordings at the subjects’ homes and the ST study provided 18 h of PSG sleep recordings in the clinic over two nights. Subjects took temazepam one night and a placebo the other night. The sampling frequency was 100 Hz. All data were manually annotated by experts based on the R & K scoring rules. In order to conduct a comparative experiment, we randomly selected 40 PSG recordings of 10 subjects in the SC study (SC 1, SC 5, SC 7, SC 10, SC 20, SC 21, SC 26, SC 27, SC 31, SC 51) and 10 subjects in the ST study (ST 4, ST 5, ST 10, ST 12, ST 15, ST 16, ST 18, ST 19, ST 20, ST 21) (demographic is shown in Table 4). Four classification tasks were conducted: a six-stage task with R, WA, S1, S2, S3, and S4 stages, a five-stage task where S3 and S4 in the six-stage classification task were combined into one stage, a four-stage task where S1 and S2 in five-stage classification were merged, and a three-stage classification only considering WA, Non-REM (S1, S2, S3, S4) and R stages. In addition, GNDA was configured by μ=0 and δ=0.4. As summarized in Table 5, this setting of δ expands twice as large as the original data of the S1 stage.

### 4.3. RNN-Based Attention Model

**Figure 7:**
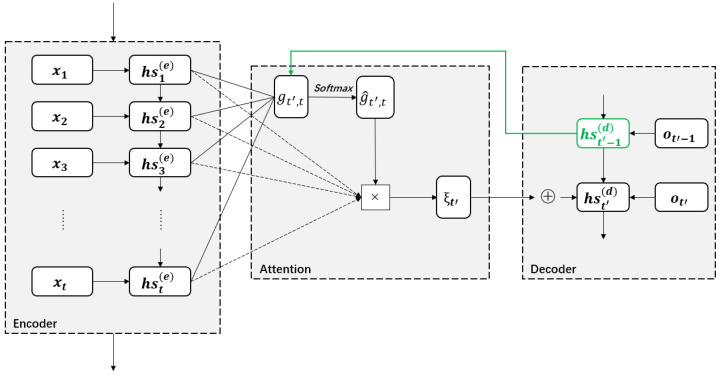
An overview of the RNN-based attention model.

One main application of the attention mechanism is sequence-to-sequence based on the encoder-decoder framework. Here, the encoder converts an input sequence x of length *T* into a context vector ξ, which summarizes the input information and is then converted into an output sequence by the decoder. The encoder and decoder are usually constructed using LSTM. The encoder’s output at timestamp *t* corresponds to a hidden state vector ${\mathrm{hs}}_{t}^{\left(e\right)}$, and the last hidden state is regarded as a context vector (i.e., $\xi ={\mathrm{hs}}_{T}^{\left(e\right)}$). However, it cannot represent the input sequence so well. The use of the attention mechanism is thus necessary to form a different context vector for each output of the decoder. The correlation $g_{t^{\prime },t}$ between the hidden state ${\mathrm{hs}}_{t^{\prime }-1}^{\left(d\right)}$ at timestamp $t^{\prime }-1$ in the decoder and all hidden states ${\left\{{\mathrm{hs}}_{t}^{\left(e\right)}\right\}}_{t=1}^{T}$ in the encoder is computed by a function $\zeta_{align}$ that attempts to capture the alignment $g_{t^{\prime },t}=\zeta_{align}\left({\mathrm{hs}}_{t^{\prime }-1}^{\left(d\right)},{\mathrm{hs}}_{t}^{\left(e\right)}\right)$ between the hidden states at timestamps *t* and $t^{\prime }-1$. The normalized attention weight ${\hat{g}}_{t^{\prime },t}$ is obtained by applying the softmax function to ${\left\{g_{t^{\prime },t}\right\}}_{t=1}^{T}$ computed for ${\left\{{\mathrm{hs}}_{t}^{\left(e\right)}\right\}}_{t=1}^{T}$. Each of these hidden states are weighted by ${\hat{g}}_{t^{\prime },t}$ and summed to form the context vector $\xi_{t^{\prime }}=\sum_{t=1}^{T}{\hat{g}}_{t^{\prime },t}{\mathrm{hs}}_{t}^{\left(e\right)}$. This way, each context vector can be associated with all hidden states in the encoder by attention. Then, $\xi_{t^{\prime }}$ collaborates with the previous hidden state ${\mathrm{hs}}_{t^{\prime }-1}^{\left(d\right)}$ in the decoder to form the hidden state ${\mathrm{hs}}_{t^{\prime }}^{\left(d\right)}$at timestamp $t^{\prime }$. The working pipeline of the RNN-based attention model is shown in Figure 7 and its implementation details are provided in Table 6.

### 4.4. Experimental Setup

Our experimental models were implemented using the *Python Keras* framework with a *Tensorflow* backend. The *Adam* optimizer was configured with learning rates of 0.001 for the RNN-based attention model and 0.0001 for DCNN and DCSAM. The batch size was set to 128 for all models, 400 epochs were used to train the RNN-based attention model, and 300 epochs were used for DCNN and DCSAM. All hyperparameters were selected by grid search [70], but also in terms of parameter traversal results from the previous study [5] since the proposed DCSAM was optimized on DCNN. Values in $\left\{16,32,64,128\right\}$ and $\left\{16,32,44\right\}$ were tested for the number of kernels in CNN and DCNN layers of DCSAM, respectively. The number of neurons in dense layers was tested with values in $\left\{440,800,1000\right\}$. The selection range from 1 to 30 with intervals of 5 was used to test the number of attention heads. For the comparative study, the number of CuDNNLSTM units in the RNN-based attention model was tested in a value range of 3 to 200 with intervals of 1. The range of values from 10 to 1000 with intervals of 10 was tested to select the number of neurons in dense layers. The grid search experiment was performed on three GPU machines in a configuration with SF=5 Hz with 150 epochs, each epoch taking about 3 s to train the DCSAM model, about 6 s to train the RNN-based attention model, and about 5 s to train the self-attention model. Three GPU machines equipped with Intel i7-8700K CPU, 128 GB RAM and two NVIDIA RTX3080Ti GPU, Intel i9-12900KF CPU, 64 GB RAM, and one NVIDIA RTX3090 24 GB GPU, and AMD Ryzen Threadripper 64Core CPU, 256 GB RAM and two NVIDIA RTX3090 24 GB GPU were used, respectively. Furthermore, we attempted to utilize a state-of-the-art approach, i.e., Support Vector Machine (SVM) with the parameters (C = 2, kernel = ’rbf’, gamma = ’scale’, max_iter = −1, decision_function_shape = ’ovr’, break_ties = True) collaborated with Discrete Wavelet Transform (DWT)-based hand-crafted features (e.g., approximation and detail coefficients of DWT for Daubechies-order wavelets—db2, db4, db6, db8, db10, db12, db14, db16, db18, and db20) [71,72] to fairly compare the performances with our proposed attention-based models.

### 4.5. Performance Evaluation on the SDCP Dataset

Table 7 and Table 8 show the performance metrics of all models configured with time window lengths of T=30 s and T=300 s, respectively. Each model has been tested under the condition that the sampling frequency was 50 Hz (performance metrics using all models based on other subsampling frequencies are shown in Appendix B). All results were evaluated using overall accuracies and F1 scores. While an overall accuracy only reflects the proportion of correctly classified samples in the whole sample set, a macro F1-score is the average of harmonic means of precision and recall that are independently calculated for each class, as depicted in the following equations:

(11) $$F1_{i}=\;\frac{2*(Precision*Recall)}{Precision+Recall}$$

(12) $$MF1=\;\frac{1}{\lambda }\sum_{i=1}^{\lambda }F1_{i},$$

where λ represents the number of sleep stages. The advantage of the macro F1-score is to treat all classes equally irrespective of the fact that the distribution of each sleep stage is unequal. To better measure the generalization of DCSAM, we performed subject-dependent seven-fold cross-validation, in which the sleep data of all subjects were mixed together and leave-one-subject-out cross-validation. All results in Table 7, Table 8 and Table A4 are based on the 7-fold cross-validation, and performances in Table 9 are based on the leave-one-subject-out cross-validation.

As shown in Table 7, the highest performance is achieved by our proposed GNDA-based DCSAM with T=30 s, SF=50 Hz, and δ=(0.2,0.4,0.6). Its accuracy (90.26%) and macro F1-score (86.51%) have improvement margins of 12.01% and 15.68% compared to the self-attention without GNDA. Additionally, the macro F1-scores of these models for the N1 and REM stages have differences of 27.95% and 14.04%. Focusing on the performances obtained by the same δ of GNDA, almost all models using GNDA have higher performances than those without its use. In addition, the performances of our DCSAM using three δs (0.2,0.4,0.6) were on average ACC of 2% higher than those only applying a single δ (0.4) and two δs (0.2,0.4). Furthermore, GNDA-based DCSAM based on non-overlapping window segmentation is more effective than that based on overlapping window segmentation. Especially, as shown in the red in Table 7 and Table 8, their notable margin in macro F1-scores is 2.94%. This further proves that non-overlapping segmentation contributes positively to DCNN to mine latent features and for the self-attention mechanism to explore the correlation between these potential features.

Moreover, in Table 7, compared to non-GNDA-based DCNN in our previous study [5], GNDA-based DCSAM achieves an improvement in accuracy and macro F1-score of 13.13% and 23.49%, respectively. This is attributed to the solution of the class imbalance problem and the analysis of correlations between features by the self-attention mechanism. In particular, for the identification of the N1 stage, there was a significant improvement of 50.41% and reach 69.2%. This is also impressive when compared to the state-of-the-art machine learning approach of DWT wavelet feature-based SVM without GN and with GN δ = (0,2,0,4,0,6), which achieved only 31.59% and 44.48% recognition sensitivity for the sleep transition stage, respectively.

Last but not least, leave-one-subject-out cross-validation based on the best configuration that can achieve the highest performance (SF=50 Hz and T=30 s) was performed to avoid classification variance caused by the increased weight in the augmented N1 stage. As can be seen in Table 9, all models achieve a stable performance compared to the subject-dependent 7-fold cross-validation (as shown in Table 7) and the DCSAM still outperforms the other models regardless of whether GNDA is used or not. With respect to the DCSAM with GN δ = (0.2,0.4,0.6), the differences in accuracy and MF1 between subject-dependent 7-fold cross-validation and leave-one-subject-out cross-validation are 3.25% and 2.51%, respectively. In particular, a small sensitive margin in the detection of the N1 stage (2.72%) further confirms the reliability of the proposed GNDA-based DCSAM.

### 4.6. Comparative Experiment on the Sleep-EDFX Dataset

To highlight the general efficiency of our proposed GNDA-based DCSAM, we performed the comparative experiment on the Sleep-EDFX dataset, leaving the hyper-parameters of the model unchanged and deploying T=30 s and the original sampling frequency SF=100 Hz with leave-one-subject-out cross-validation. Due to the difference in the data scale distributions of the SDCP and Sleep-EDFX datasets, we changed δ in GNDA from (0.2,0.4,0.6) to 0.4 and apply GNDA to the training folds during cross-validation. In addition, an *Adamax* optimizer with a learning rate of 0.002 was used in this experiment. Table 10 summarizes the performances of GNDA-based DCSAM using single-channel EEG (*Fpz-Cz*) with δ=0.4 for the six-stage to three-stage scoring tasks. Our model can obtain strong performances on these tasks with average accuracies of 91.77%, 92.54%, 94.73%, and 95.30%, and average macro F1-scores of 86.64%, 88.85%, 91.41%, and 93.01%, respectively.

**Figure 8:**
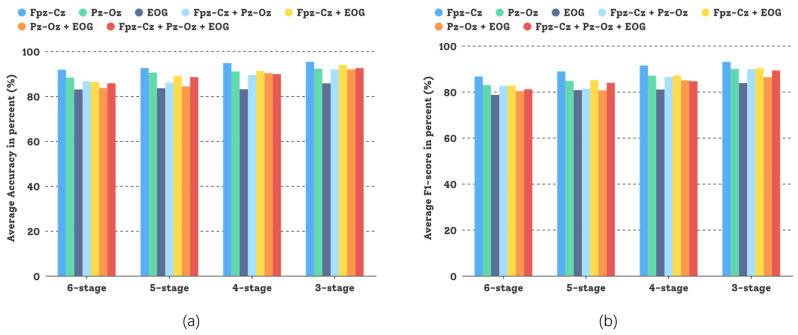
Performance comparison of our GNDA-based DCSAM using different sensor channels in the three-stage to the six-stage classification of sleep stage on the Sleep-EDFX dataset (T=30 s, SF=100 Hz, and δ=0.4): (**a**) Average overall accuracy comparison; (**b**) Average F1-score comparison.

In this comparative study, different sensor modalities and channels were tested and the experimental results are shown in Figure 8. Both sensor modalities, *Fpz-Cz* and *Pz-Oz*—and even their combination—could attain good experimental results, but for the Sleep-EDFX dataset, using a single channel based on the DCSAM is more effective than using multiple channels, and the investment in low noise (small random variation) pays off. This demonstrates the generalization capability of our GNDA-based DCSAM.

### 4.7. Discussion

First of all, the success of the experiments should be attributed to GNDA. As shown in Table 3, the proportion of the N1 stage has only 5.76%, it is so small that the models cannot fully learn the features of this stage. The expansion of the N1 proportion allows a better interpretation of the characteristics of this sleep stage by the self-attention mechanism, distinguishing it well from the REM stage. Regardless of the model, the results of using GNDA are better than not applying it (as shown in Table 7, Table 8 and Table 9).

**Figure 9:**
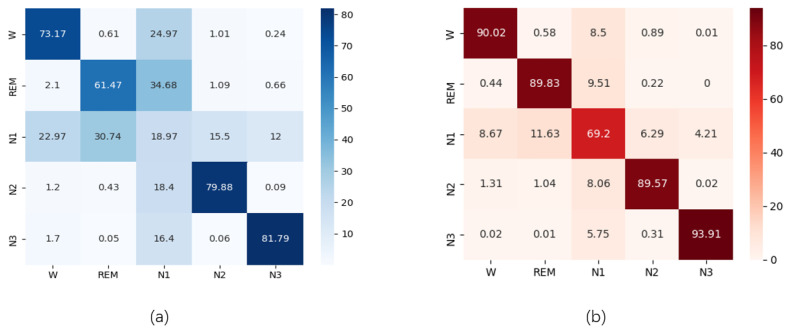
Performance comparison in the form of a confusion matrix: (**a**) Confusion matrix based on the SWS-based DCNN without GNDA using *T* = 30 s and *SF* = 50 Hz obtained from the SDCP dataset; (**b**) Confusion matrix based on GNDA-based DCSAM with T=30 s, SF=50 Hz, and δ=(0.2,0.4,0.6) obtained from the SDCP dataset.

**Figure 10:**
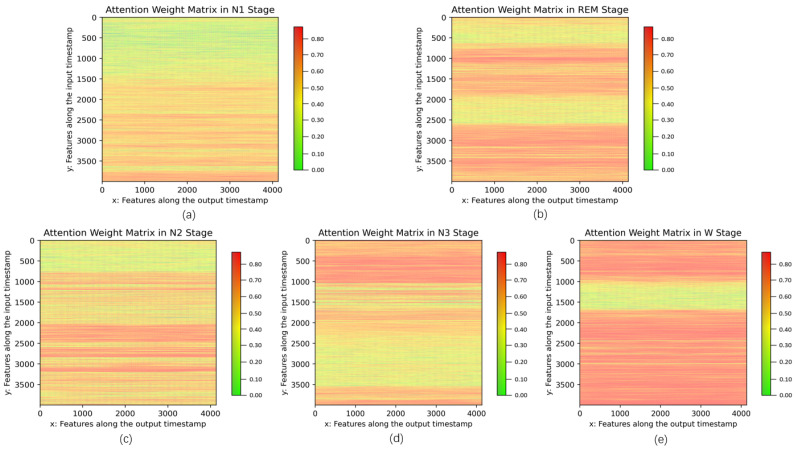
Visualization of attention weights after using GNDA-based DCSAM with multiple sensor channels on the SDCP dataset (T=30 s, SF=50 Hz). Attention weights (the correlations between features) are presented at each sleep stage, respectively: (**a**) N1; (**b**) REM; (**c**) N2; (**d**) N3; (**e**) W.

Next, according to the principle of the self-attention mechanism, the specificity of the features in the N1 stage can be better distinguished in context, which greatly reduces the misclassification risk between the N1 and other sleep stages as depicted in Figure 9. Figure 10 illustrates the qualitative analysis results of self-attention weights for five sleep stages. The self-attention mechanism computes attention weights ${\hat{g}}_{t,t^{\prime }}$ utilizing the softmax function following Equation (6). A matrix formed by collecting these attention weights emphasizes the influence of an input temporal feature on a higher-level output temporal feature. Since each self-attention head can focus on distinct features, the attention weight matrices based on 15 self-attention heads were averaged and visualized by depicting high and low attention weights in orange and green, respectively. The attention weights are exhibited as vertical lines corresponding to the values in ${\hat{g}}_{t,t^{\prime }}$. To intuitively observe the difference in attention weights for different sleep stages, we averaged the attention weight matrices for a certain class to create 5 class-specific attention weight matrices. Different sleep stages pay more attention to specific parts of the time series, as shown in orange areas in Figure 10. For instance, the N1 stage appears to focus on the late-middle part of a segment while the REM stage learns the features from the early-middle and late parts. Therefore, each sleep stage is clearly distinguished from the other by taking into account the distribution of attention weights. In other words, the model focuses on informative parts of a segment, such as strong peaks corresponding to functional subbands, such as K-complex and spindles. This demonstrates the efficiency of the self-attention mechanism for mining feature correlations.

**Figure 11:**
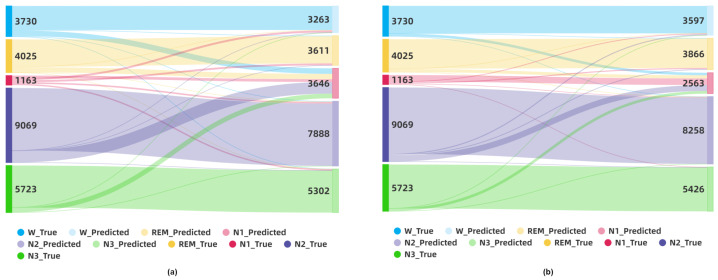
Visualization of sleep stage classification results on the SDCP dataset using the Sankey diagram: (**a**) Sankey diagram of the classification result by the non-GNDA-based DCNN using the leave-one-subject-out cross-validation; (**b**) Sankey diagram of the classification result by the GNDA-based DCSAM using the leave-one-subject-out cross-validation.

To claim this argument more closely and visually, we also created the Sankey diagrams in Figure 11 to show how the classification results change on an aggregated level. For the SDCP dataset, the numbers of ground truth labels and predicted labels are depicted by dark colors on the left side and light colors on the right side, respectively. The classification statuses in sleep stages are shown as a flow between the left and right sides. For instance, in the red in Figure 11a, there are 1163 N1 labels in the ground truth, but the number of N1 labels predicted by the non-GNDA-based DCNN using leave-one-subject-out cross-validation is 3646. This means that misclassifications between the N1 and other sleep stages are quite severe. The advantages of GNDA and self-attention mechanisms are obvious in comparison. After applying GNDA, a total of 2563 predicted labels as N1 are obtained after using DCSAM with the same cross-validation strategy, as shown in Figure 11b. Although there are still misclassifications among them, most of the true N1 labels are correctly identified, so that the red-colored visual flow from the ground truth N1 labels to the predicted ones in Figure 11b is more concentrated and less dispersed. In addition to N1, other sleep labels were correctly identified much more accurately compared to Figure 11a. This further validates that our GNDA-based DCSAM is able to effectively distinguish the N1 from the REM stage by overcoming the imbalanced class problem.

As shown in Table 7, the experimental performance of the GNDA-based DCNN with δ=(0.2,0.4,0.6) is significantly improved in MF1 by a notable margin of approximately 20.03% after it adopts the self-attention mechanism. Compared to other studies [54,56,57,73], our model could also achieve strong and stable performance and even better accuracies and macro F1-scores for the recognition of the sleep transition stage. Meanwhile, non-overlapping segmentation (T=30 s) is beneficial for the self-attention mechanism to exploit the correlation between features of different sleep stages. On the other hand, the determination of representative labels is more difficult in overlapping segments (T=300 s), and the incorrect selection of representative annotations can lead to inter-class misclassifications. This also makes the self-attention mechanism misunderstand relevant features and their correlations.

**Figure 12:**
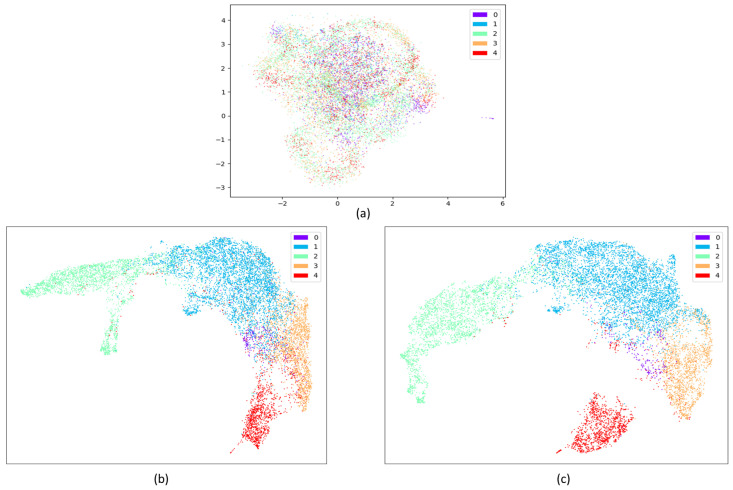
Visualization of the raw data distribution on the SDCP dataset, as well as visualizations of the features obtained from the last DCNN layer and the last attention layer using UMAP [74] (0: N1 stage; 1: N2 stage; 2: N3 stage; 3: REM stage; 4: W stage). (**a**) Visualization of the raw data distribution on the SDCP dataset; (**b**) Visualization of the features obtained from the last DCNN layer; (**c**) Visualization of the features obtained from the last attention layer.

Overall, the contribution of the attention mechanism compared to the DCNN is also evident when analyzed at the mechanism level. Figure 12 shows the visualization of the raw data distribution on the SDCP dataset, as well as visualizations of the features obtained from the last DCNN layer and the last attention layer to intuitively demonstrate the effectiveness of the core attention mechanism. Raw data (as illustrated in Figure 12a) are usually cluttered with noise. DCNN plays an initial screening role and offers an advantage in DCSAM, having little dependence on preprocessing while reducing the human effort required for feature extraction. It automatically learns the temporal and latent features from the raw sensor data. Almost all sleep stages can be well identified, except for the N1 and REM stages, because their short duration and their features in EEG and EMG are similar to the REM stage (as shown in Figure 12b). In turn, the core attention mechanism performs inter-feature correlations analysis using attention weights, making the features of the N1 stage highly distinguishable from other sleep stages (as shown in Figure 12c), thus further improving the performance of the overall classification.

**Figure 13:**
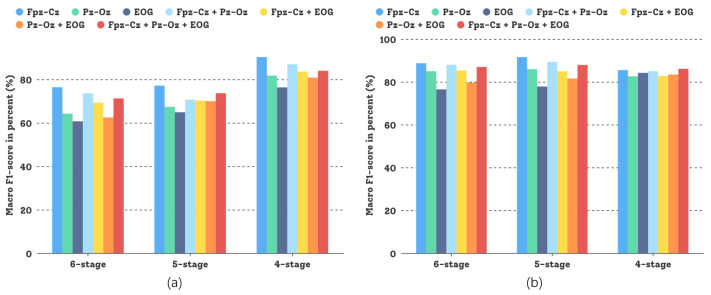
Comparison of our GNDA-based DCSAM using different sensor channels for the S1 (S1/S2) stage (**a**) and R stage (**b**) on the Sleep-EDFX dataset (T=30 s, SF=100 Hz, and δ=0.4).

In the Sleep-EDFX dataset, as shown in Table 5, the proportion of the S1 stage is 5.58%, and the difference is not very large compared to the R stage. Therefore, there is no need to expand data for the S1 stage too much, and adding Gaussian noise based on a single δ can improve the results obviously. As shown in Figure 13, the features of the S1 and R stages can be efficiently learned regardless of which sensor channel is used and the recognition sensitivity of S1 and R stages for the six-stage sleep classification can reach 76.40% and 88.69%, respectively. In addition, compared to other studies [12,36,37,38,39,40,45,47,52,73,75], which has performed feature engineering to generate hand-crafted features in an EEG signal, such as wavelet transform, spectral entropy, and time-frequency image, we deal with raw multi-channel PSG recordings and uses the deconvolutional block and self-attention mechanism to exploit the latent features and their correlations. The performance comparison between our GNDA-based DCSAM and other state-of-the-art methods is shown in Table 11. The bias of the results depends on the hyperparameter settings of the specific experimental model. We use accuracy as the main evaluation metric for this comparison because it is used in most studies. In Table 11, TFA+SSAE [76] and DWT+MSPCA+RotSVM [40] based on feature engineering with single-channel EEG achieve the highest accuracies of 82% and 91.10% for the five-stage classification task, respectively. CNN-Att [54], which adopts an attention mechanism, attained the highest accuracy of 93.7% for the five-stage classification task. In contrast, our model with a single-channel EEG achieved an average accuracy of 91.77% to 95.30%, depending on how many sleep classes are considered. This performance is comparable to those of the top-ranked methods in Table 11, which indicates the great potential of our model.

All the performances prove the validity and plasticity of our model as it contributes to the classification of sleep stages in children by investigating a strategy of data balance and self-attention mechanism to improve the accuracy of sleep transition detection. In general, our proposed DCSAM can also be used for timestamp-based classification tasks for multivariate time series in various medical fields.

## 5. Conclusions

In this paper, we proposed a *DeConvolution and Self-Attention-based Model* (DCSAM) with *Gaussian Noise Data Augmentation* (GNDA) using multi-channel PSG recordings to address sleep stage classification for child patients. Compared to our previous study, we inserted a multi-head self-attention mechanism in the *DeConvolutional Neural Network* (DCNN) to dig deeper into the correlation between all possible feature pairs extracted by convolutional and deconvolutional blocks to accurately distinguish sleep stages from each other. Meanwhile, GNDA was used to expand the proportion of the sleep transition stage N1, so that more opportunities were offered for DCSAM to learn better features of this stage. GNDA is an important challenge to improving the performance of minority classes such as N1 without feature engineering. We also conducted comparative experiments using our proposed GNDA-based DCSAM on the Sleep-EDFX dataset and proved its stable performances.

To further optimize our GNDA-based DCSAM, we plan to investigate the following issues: To balance the class, we will first try to use the dual-pipeline mechanism of *Robust Conditional Generative Adversarial Network* (RoCGAN) [81] to simulate sleep data at the N1 stage and expand the data, or we will attempt to implement ensemble and transfer learning [82,83], i.e., use relevant knowledge about sleep from training a large public dataset to facilitate the learning of sleep features in a new dataset. The data-driven DNN models are widely used to classify sleep stages and can achieve a reasonable performance. This is expected to significantly reduce the reliance on manual labeling. However, the problem of domain shift usually occurs in real applications. Therefore, to prevent the loss of domain-specific information during feature extraction and to align the fine-grained class distributions for the source and target domains via pseudo-labels of the target domain, we can develop an *Unsupervised Domain Adaption* (UDA)-based [84] unshared attention mechanism that uses an iterative self-training strategy to solve this domain-shift problem in the unlabeled target domain. In addition, video, accelerometer, and gyroscope data collected by *Microsoft Kinect* and *Inertial Measurement Unit* (IMU) [85] can be used to track a child’s body movements during sleep.

## Figures and Tables

**Table 1:** The architecture and hyper-parameters of the proposed DCSAM.

Layer Number	Layer Type	Parameter	Activation Function	Value
1	conv.	# kernels Sliding stride size Kernel size	LeakyRuLU same-padding -	16 (1, 1) (4, 1)
2	BatchNormalization	-	LeakyReLU	-
3	conv.	# kernels Sliding stride size Kernel size	LeakyRuLU same-padding -	16 (1, 1) (3, 1)
4	max-pooling	pooling size pooling stride size	- -	(3, 3) (1, 1)
5	conv.	# kernels Sliding stride size Kernel size	LeakyRuLU same-padding -	32 (1, 1) (2, 1)
6	max-pooling	pooling size pooling stride size	- -	(3, 3) (2, 1)
7	conv.	# kernels Sliding stride size Kernel size	LeakyRuLU same-padding -	64 (1, 1) (4, 1)
8	max-pooling	pooling size pooling stride size	- -	(3, 3) (2, 1)
9	conv.	# kernels Sliding stride size Kernel size	LeakyRuLU same-padding -	128 (1, 1) (5, 1)
10, 11	deconv.	# kernels Sliding stride size Kernel size	LeakyRuLU valid-padding -	16, 44 (1, 1), (1, 1) (1, 1), (3, 1)
12	GAP	-	-	-
13	positional embedding	-	-	-
14	attention mechanism	*num-head* *dropout rate* *attention-axes* *Q,K,V*	- - - -	H = 15 0.5 None T x HD
15	add & normalization	-	-	-
16	fully-connected	# neurons	ReLU	440
17	add & normalization	-	-	-
18	fully-connected	# neurons	ReLU	800
19	Dropout	drop rate	-	0.3
20	softmax	# neurons	logistic	5

**Table 2:** Demographic of 21 subjects in the SDCP dataset.

Subject	Age	Sex	Night (Lights Off)	Subject	Age	Sex	Night (Lights Off)
P 1	6 years old	female	20:43:27	P 13	4 years old	male	19:53:15
P 2	5 years old	female	20:54:01	P 14	10 years old	female	22:13:41
P 3	6 years old	male	22:07:31	P 15	8 years old	male	21:52:36
P 4	7 years old	male	21:07:10	P 16	5 years old	female	19:36:53
P 5	10 years old	female	22:29:08	P 17	10 years old	female	21:32:44
P 6	8 years old	female	21:42:40	P 18	6 years old	male	20:30:44
P 7	9 years old	female	20:51:50	P 19	5 years old	female	20:12:13
P 8	7 years old	male	21:34:06	P 20	6 years old	female	21:36:07
P 9	5 years old	male	21:01:43	P 21	7 years old	female	20:42:59
P 10	4 years old	female	20:55:15	P 24	7 years old	female	20:48:00
P 11	5 years old	female	22:19:57				

**Table 3:** Proportional changes of the sleep stage by applying GNDA to data of the N1 stage in the training set (17 subjects) in our SDCP dataset (#segments of sleep stages (%)). μ and δ represent the mean and the standard deviation of a Gaussian distribution, respectively. The values of the proportion before and after applying GNDA are highlighted in green and red, respectively.

	N1	N2	N3	REM	W
Original	1113 (5.76)	6805 (35.24)	4743 (24.56)	3288 (17.03)	3361 (17.41)
μ = 0, δ = 0.4	2226 (10.90)	6805 (33.32)	4743 (23.22)	3288 (16.10)	3361 (16.46)
μ = 0, δ = 0.2, 0.4	3339 (15.50)	6805 (31.60)	4743 (22.02)	3288 (15.27)	3361 (15.61)
μ = 0, δ = 0.2, 0.4, 0.6	4452 (19.65)	6805 (30.05)	4743 (20.94)	3288 (14.52)	3361 (14.84)

**Table 4:** Demographic of 20 subjects in the Sleep-EDFX dataset.

Study/Subject	Age	Sex	Placebo Night (Lights Off)	Temazepam Night (Lights Off)
SC 1	33 years old	female	22:44	22:15
SC 5	28 years old	female	1:22	0:35
SC 7	30 years old	female	0:36	0:41
SC 10	26 years old	male	22:59	23:07
SC 20	51 years old	female	23:10	23:15
SC 21	51 years old	female	23:28	23:59
SC 26	51 years old	female	23:39	0:20
SC 27	54 years old	female	23:41	22:58
SC 31	54 years old	male	23:44	23:14
SC 51	70 years old	male	23:10	0:03
-	-	-	**Placebo Night (Lights Off)**	**Temazepam Night (Lights Off)**
ST 4	18 years old	female	23:53	22:37
ST 5	32 years old	female	23:23	23:34
ST 10	20 years old	female	23:21	23:28
ST 12	21 years old	male	23:46	23:56
ST 15	66 years old	female	23:42	23:33
ST 16	79 years old	female	23:21	23:18
ST 18	53 years old	female	23:38	23:24
ST 19	28 years old	female	23:22	23:44
ST 20	24 years old	male	23:47	0:01
ST 21	34 years old	female	23:44	23:10

**Table 5:** Proportional changes in sleep stages by applying GNDA to data of the S1 stage in the training set (14 subjects) in the Sleep-EDFX dataset(#segments of sleep stages (%)). μ and δ represent the mean and the standard deviation of a Gaussian distribution, respectively. The values of the proportion before and after applying GNDA are highlighted in green and red, respectively.

	S1	S2	S3	S4	R	WA
Original	3203 (5.58)	13,499 (23.52)	2444 (4.26)	2201 (3.83)	5551 (9.67)	30,498 (53.14)
μ = 0, δ = 0.4	6406 (10.57)	13,499 (22.28)	2444 (4.03)	2201 (3.63)	5551 (9.16)	30,498 (50.33)

**Table 6:** The architecture of the RNN-based attention model.

Layer Number	Layer Type	Parameter	Activation Function	Value
1	positional embedding	-	-	-
2	LSTM cell	# units dropout recurrent_dropout	tanh - -	110 0.5 0.3
3	attention mechanism	-	-	-
4	fully-connected	# neurons	ReLU	380
5	dropout	drop rate	-	0.2
6	softmax	# neurons	logistic	5

**Table 7:** Final performance metrics of all models with and without GNDA based on the SWS strategy (ΔS is fixed at 30 s) with SF=50 Hz and T=30 s, and 7-fold cross-validation on the SDCP dataset (Macro F1-Score = MF1, Accuracy = ACC, Gaussian Noise Data Augmentation = GNDA, GNDA was applied only to the training folds during cross-validation; the highest performances are highlighted in red).

	50 Hz		MF1 for Each Class				
	ACC	MF1	N1	N2	N3	REM	W
DWT + SVM without GNDA	67.59	55.52	31.59	64.20	66.92	57.14	57.75
GNDA(0.4) + DWT + SVM	67.81	52.05	29.97	62.54	64.69	55.49	47.56
GNDA(0.2, 0.4) + DWT + SVM	71.12	56.44	37.19	67.54	60.22	58.75	58.50
GNDA(0.2, 0.4, 0.6) + DWT + SVM	71.97	59.45	44.48	69.36	72.11	60.98	50.32
DCNN without GNDA	77.13	63.02	18.79	79.88	81.79	61.47	73.17
GNDA(0.4) + DCNN	80.01	66.88	25.47	82.09	82.19	69.56	75.09
GNDA(0.2, 0.4) + DCNN	80.34	67.35	26.43	81.84	82.29	72.63	73.53
GNDA(0.2, 0.4, 0.6) + DCNN	79.52	66.48	23.92	80.23	83.66	71.62	72.96
RNN-based attention without GNDA	71.48	65.27	29.97	77.67	80.09	69.68	68.94
GNDA(0.4) + RNN-based attention	73.98	66.47	32.58	78.39	81.80	71.59	67.99
GNDA(0.2, 0.4) + RNN-based attention	71.57	63.99	31.69	77.98	79.77	67.43	63.08
GNDA(0.2, 0.4, 0.6) + RNN-based attention	74.68	68.24	33.34	80.17	81.62	71.87	74.20
Self-attention without GNDA	78.25	70.83	41.25	82.00	84.27	75.89	70.74
GNDA(0.4) + Self-attention	82.97	75.87	46.84	84.08	86.95	81.24	80.24
GNDA(0.2, 0.4) + Self-attention	84.45	77.75	47.88	85.97	88.05	83.00	83.85
GNDA(0.2, 0.4, 0.6) + Self-attention	82.67	75.87	46.14	85.00	86.29	79.40	82.52
GNDA(0.4) + DCNN + Self-Attention	87.37	85.22	67.15	87.00	90.87	89.26	91.82
GNDA(0.2, 0.4) + DCNN + Self-Attention	88.55	84.69	66.72	86.17	91.89	90.44	88.23
GNDA(0.2, 0.4, 0.6) + DCNN + Self-Attention	90.26	86.51	69.20	89.57	93.91	89.83	90.02

**Table 8:** Final performance metrics of all models with and without GNDA based on the SWS strategy (ΔS is fixed at 30 s) with SF=50 Hz and T=300 s, and 7-fold cross-validation on the SDCP dataset (Macro F1-Score = MF1, Accuracy = ACC, Gaussian Noise Data Augmentation = GNDA, GNDA was applied only to the training folds during cross-validation; the highest performances are highlighted in bold).

Model	50 Hz		MF1 for Each Class				
	ACC	MF1	N1	N2	N3	REM	W
DWT + SVM without GNDA	67.01	53.79	26.75	60.20	62.00	50.07	69.93
GNDA(0.4) + DWT + SVM	68.92	52.97	27.15	61.13	60.49	48.04	68.04
GNDA(0.2, 0.4) + DWT + SVM	69.92	52.99	29.13	60.89	62.05	49.91	62.97
GNDA(0.2, 0.4, 0.6) + DWT + SVM	69.94	54.18	29.99	62.05	61.79	51.52	65.55
DCNN without GNDA	77.90	66.11	26.93	84.77	86.18	63.79	68.88
GNDA(0.4) + DCNN	80.67	71.39	32.78	85.12	88.26	70.68	83.08
GNDA(0.2, 0.4) + DCNN	86.02	75.88	36.03	86.17	89.21	80.57	87.42
GNDA(0.2, 0.4, 0.6) + DCNN	85.81	76.11	35.07	86.11	89.00	82.83	87.54
RNN-based attention without GNDA	71.03	64.51	28.57	76.18	80.91	68.23	68.04
GNDA(0.4) + RNN-based attention	70.44	63.06	29.67	77.26	78.66	65.76	63.95
GNDA(0.2, 0.4) + RNN-based attention	68.76	62.28	27.99	75.98	76.00	66.90	64.53
GNDA(0.2, 0.4, 0.6) + RNN-based attention	70.56	66.47	30.71	79.98	81.22	71.00	69.44
Self-attention without GNDA	77.29	68.27	39.69	80.01	83.25	74.20	67.20
GNDA(0.4) + Self-attention	80.67	73.88	43.57	83.55	86.29	77.41	78.58
GNDA(0.2, 0.4) + Self-attention	80.05	72.97	44.30	80.95	83.98	76.62	79.00
GNDA(0.2, 0.4, 0.6) + Self-attention	81.15	74.19	44.08	81.54	85.36	78.18	81.79
GNDA(0.4) + DCNN + Self-Attention	86.24	81.89	61.78	86.24	88.89	86.97	85.57
GNDA(0.2, 0.4) + DCNN + Self-Attention	88.06	83.18	65.34	84.77	89.09	90.16	86.54
GNDA(0.2, 0.4, 0.6) + DCNN + Self-Attention	**88.56**	**83.57**	**66.05**	85.42	90.71	87.45	88.22

**Table 9:** Performance metrics of all models with and without GNDA based on the SWS strategy (ΔS is fixed at 30 s) with SF=50 Hz and T=30 s, and leave-one-subject-out cross-validation on the SDCP dataset (Macro F1-Score = MF1, Accuracy = ACC, Gaussian Noise Data Augmentation = GNDA, GNDA was applied only to the training folds during cross-validation; the highest performances are highlighted in red).

	50 Hz		MF1 for Each Class				
	ACC	MF1	N1	N2	N3	REM	W
DWT + SVM without GNDA	65.00	50.03	26.10	61.75	63.49	53.02	45.79
GNDA(0.4) + DWT + SVM	63.37	46.67	24.89	57.86	60.05	50.49	40.06
GNDA(0.2, 0.4) + DWT + SVM	66.62	50.28	30.49	60.57	63.25	51.70	45.39
GNDA(0.2, 0.4, 0.6) + DWT + SVM	68.07	52.88	35.34	62.19	64.86	55.57	46.44
DCNN without GNDA	74.73	59.49	16.30	77.54	80.69	56.30	66.62
GNDA(0.4) + DCNN	77.34	63.99	22.90	80.06	80.94	61.98	74.07
GNDA(0.2, 0.4) + DCNN	78.65	64.02	24.04	82.53	80.47	60.37	72.69
GNDA(0.2, 0.4, 0.6) + DCNN	76.54	62.70	21.94	80.50	80.40	63.01	67.65
RNN-based attention without GNDA	69.60	64.03	27.00	78.63	79.40	67.23	67.89
GNDA(0.4) + RNN-based attention	70.45	64.87	30.59	79.03	80.33	69.99	64.41
GNDA(0.2, 0.4) + RNN-based attention	69.03	60.51	28.43	76.35	78.00	64.33	55.44
GNDA(0.2, 0.4, 0.6) + RNN-based attention	71.28	66.36	29.58	80.04	79.21	70.46	72.51
Self-attention without GNDA	77.62	69.40	38.97	81.91	82.67	73.90	69.55
GNDA(0.4) + Self-attention	79.45	73.60	43.82	83.98	84.06	79.28	76.86
GNDA(0.2, 0.4) + Self-attention	83.06	75.24	45.00	83.97	86.59	81.29	79.35
GNDA(0.2, 0.4, 0.6) + Self-attention	82.00	74.32	44.66	82.69	84.37	77.14	82.74
GNDA(0.4) + DCNN + Self-Attention	85.07	83.24	65.83	85.26	88.40	88.14	88.57
GNDA(0.2, 0.4) + DCNN + Self-Attention	85.86	83.09	64.70	85.09	89.26	90.00	86.40
GNDA(0.2, 0.4, 0.6) + DCNN + Self-Attention	86.91	84.00	66.48	86.44	90.39	87.69	89.00

**Table 10:** The performance of our GNDA-based DCSAM using leave-one-subject-out cross-validation (T=30 s, SF=100 Hz, δ=0.4), *Fpz-Cz* on the Sleep-EDFX dataset. Average F1-Score = AF1, Average Accuracy = AACC, GNDA is applied only to the training folds during cross-validation, and the highest performances are highlighted in red.

	AF1	AACC	Average F1-Score of Each Sleep Stage					
			WA	S1	S2	S3	S4	R
6-stage	86.64	91.77	92.79	76.40	87.94	82.07	91.94	88.69
5-stage	88.85	92.54	92.99	77.14	89.39	(S3/S4: 93.16)		91.57
4-stage	91.41	94.73	94.01	(S1/S2: 90.29)		(S3/S4: 95.83)		85.51
3-stage	93.01	95.30	94.05	(S1/S2/S3/S4: 98.47)				86.51

**Table 11:** Performance comparison of various state-of-the-art methods on the Sleep-EDFX dataset with 20 subjects. Our proposed method is highlighted in bold. TFA: Time-frequency Analysis; SSAE: Stacked Sparse Autoencoder; CNN: Convolutional Neural Network; BiLSTM: Bidirectional-LSTM; DWT: Discrete Wavelet Transform; SVM: Support Vector Machine; MSPCA: Multiscale Principal Component Analysis; RotSVM: Rotational Support Vector Machine; MT-CNN: Multi-task CNN; CNN-Att: CNN-based attention model; MB-CNN: Multi-Branch Convolutional Neural Network; MS-DAN: Multi-scale Dual Attention Network; OC-SVM: One-class SVM; MRCNN: Multi-resolution convolutional neural network; AFR: Adaptive feature recalibration; TCE: Temporal context encoder.

Study	Dataset & Subjects	Channel	Performance			
			Overall Accuracy (%)			
			6-Stage	5-Stage	4-Stage	3-Stage
TFA+SSAE [76]	Sleep-EDFX	Fpz-Cz	-	82.00	-	-
CNN+BiLSTM [52]	Sleep-EDFX	Fpz-Cz	-	82.00	-	-
DWT+MSPCA+RotSVM [40]	Sleep-EDFX	Pz-Oz	-	91.10	-	-
1D-CNN [12]	Sleep-EDFX	Fpz-Cz +EOG	89.54	90.98	92.33	94.34
MT-CNN [77]	Sleep-EDFX	Fpz-Oz + EOG	-	82.30	-	-
CNN-Att [54]	Sleep-EDFX	Fpz-Cz	-	93.7	-	-
MB-CNN [78]	Sleep-EDFX	Fpz-Cz + Pz-Oz + EOG	-	85.80	-	-
MS-DAN [79]	Sleep-EDFX	Fpz-Cz	-	90.35	-	-
SVM+ OC-SVM [80]	Sleep-EDFX	Fpz-Cz + Pz-Oz	93.00	93.40	-	-
MRCNN+AFR+TCE [73]	Sleep-EDFX	Fpz-Cz	-	85.6	-	-
CNN+LSTM [13]	Sleep-EDFX	Fpz-Cz + Pz-Oz + EOG	-	87.50	-	-
Proposed method	Sleep-EDFX	Fpz-Cz + Pz-Oz + EOG	85.75	88.50	89.81	92.52
Proposed method	Sleep-EDFX	Pz-Oz	88.24	90.51	91.02	92.22
Proposed method	Sleep-EDFX	Fpz-Cz + Pz-Oz	86.73	86.00	89.42	91.83
Proposed method	Sleep-EDFX	Fpz-Cz + EOG	86.40	88.99	91.30	93.86
**Proposed method**	**Sleep-EDFX**	**Fpz-Cz**	**91.77**	**92.54**	**94.73**	**95.30**

**Figure A1:**
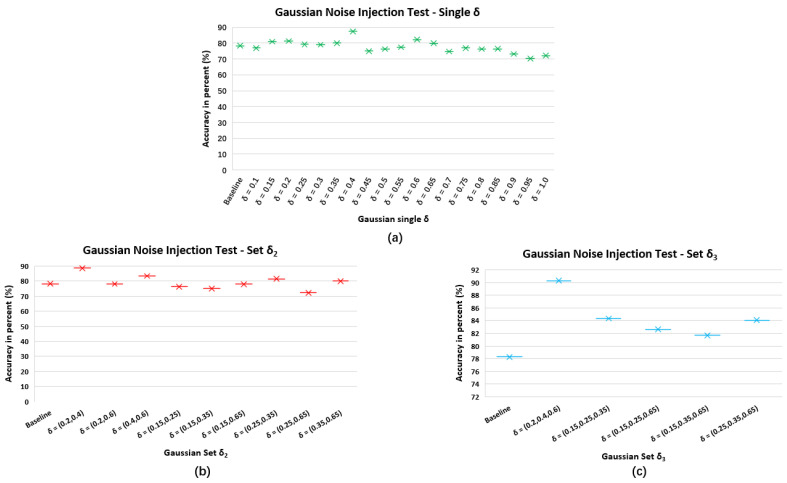
Performance comparison between the baseline model (non-GNDA-based DCNN) and pretrained DCSAM with different GN δ settings: (**a**) train on Gaussian noise selected from a distribution with a single δ; (**b**) train on Gaussian noise distribution where δ is selected from Gaussian standard deviation set $\delta_{2}$; (**c**) train on Gaussian noise distribution where δ is selected from Gaussian standard deviation set $\delta_{3}$.

## Data Availability

The Sleep-EDFX dataset is available at https://www.physionet.org/content/sleep-edfx/1.0.0/ (accessed on 27 February 2023). Further data sharing is not applicable to this article.

## Abbreviations

The following abbreviations are used in this article:

GNDA	Gaussian Noise Data Augmentation
DCSAM	DeConvolution- and Self-Attention-based Model
PSG	Polysomnography
R & K	Rechtschaffen & Kales
AASM	American Academy of Sleep Medicine
REM	Rapid Eye Movement
EEG	Electroencephalography
EOG	Electrooculography
EMG	Electromyography
PPG	Photoplethysmogram
NLP	Natural Language Processing
CV	Computer Vision
TSA	Time Series Analysis
DWT	Discrete Wavelet Transform
SVM	Support Vector Machine
DCNN	DeConvolutional Neural Network
Bi-LSTM	Bidirectional Long Short-term Memory
CNN	Convolutional Neural Network
LeakyReLU	Leaky Rectified Linear Unit
FT	Fourier Transform
SFA	Spectral Features Analysis
TA	Time-frequency Analysis
LAMF	Low Amplitude Mixed Frequency
SWS	Sliding Window Segmentation
JS	Jacobian Score
GAN	Generative Adversarial Networks
IMU	Inertial Measurement Unit
Acc	Accuracy
MF1	Macro F1 score

## Appendix A. Gaussian Noise Injection Test

This appendix presents the *Gaussian Noise* (GN) injection test to show which GN characterized by standard deviation δ is efficient for our experiments. We adopt a pretrained DCNN in our previous study [5] as a baseline and then train GNDA-based DCSAM using grid search to find the optimal δ. As illustrated in Figure A1, three different settings were compared: First, a single GN δ characterized by 19 equidistant values from [0,1] with intervals of 0.05 was tested as shown in the green points in Figure A1a. The second and third δs are selected based on the performance of the single GN δ test, which allows better accuracy of the DCSAM with GNDA than the baseline. They can build pairs $\delta_{2}$ and triples $\delta_{3}$, as shown in the red points and blue points in Figure A1b,c, respectively. We can thus intuitively select the effective GN δ combinations used for our subsequent experiments, i.e., three comparison groups of SD:δ: δ=0.4, δ=(0.2,0.4) and δ=(0.2,0.4,0.6) while the mean μ is fixed to zero.

## Appendix B. Subsampling Frequency Test

This appendix provides the performance comparison based on the subsampling frequency test to select the optimal SF for the experiment. The original sampling frequency of sleep PSG recordings is 200 Hz. For our study, according to the functional subbands in EEG, EOG, and EMG (defined in Section 4.1.2), we test three low subsampling frequencies, 5 Hz, 10 Hz, and 50 Hz. Table A1 and Table A2 show the performance metrics using all models based on T=30 s and T=300 s configured with SF=5 Hz, 10 Hz, and 50 Hz. The results are evaluated by overall accuracy and macro F1-score. We can find that the increase in the subsampling frequency has a positive effect regardless of whether it depends on the non-overlapping or overlapping segmentation. 50 Hz is determined by reconciling calculation complexity, calculation performance, and learning speed. In terms of experimental performances, the use of low subsampling frequencies in sleep stage classification is worth quantifying and investigating.

**Table A1:** Performance metrics of all models with and without Gaussian noise data augmentation based on the SWS strategy (ΔS is fixed at 30 s) with SF=5 Hz, 10 Hz, and 50 Hz and T=30 s by 7-fold cross-validation for sleep stage classification on the SDCP dataset (Macro F1-Score = MF1, Accuracy = ACC, Gaussian Noise Data Augmentation = GNDA, GNDA is applied only to the training folds during cross-validation, highest performances are highlighted in red).

Model	5 Hz		10 Hz		50 Hz	
	ACC	MF1	ACC	MF1	ACC	MF1
DWT + SVM without GNDA	52.69	45.43	56.98	48.11	67.59	55.52
GNDA(0.4) + DWT + SVM	54.40	46.02	56.50	46.86	67.81	52.05
GNDA(0.2, 0.4) + DWT + SVM	58.01	49.97	60.93	51.92	71.12	56.44
GNDA(0.2, 0.4, 0.6) + DWT + SVM	60.32	52.53	64.03	54.07	71.97	59.45
DCNN without GNDA	74.48	61.39	76.33	62.17	77.13	63.02
GNDA(0.4) + DCNN	74.92	63.23	78.56	64.71	80.01	68.88
GNDA(0.2, 0.4) + DCNN	76.57	62.82	78.19	65.14	80.34	67.35
GNDA(0.2, 0.4, 0.6) + DCNN	77.12	63.19	78.45	64.66	79.52	66.48
RNN-based attention without GNDA	69.78	63.51	70.29	63.96	71.48	65.27
GNDA(0.4) + RNN-based attention	71.33	65.48	72.55	64.28	73.98	66.47
GNDA(0.2, 0.4) + RNN-based attention	70.40	63.03	69.89	61.57	71.57	63.99
GNDA(0.2, 0.4, 0.6) + RNN-based attention	73.01	66.79	74.52	67.24	74.68	68.24
Self-attention without GNDA	76.47	68.24	77.11	68.97	78.25	70.83
GNDA(0.4) + Self-attention	80.65	71.59	82.07	74.50	82.97	75.87
GNDA(0.2, 0.4) + Self-attention	78.98	70.24	80.86	71.34	84.45	77.75
GNDA(0.2, 0.4, 0.6) + Self-attention	81.17	73.78	83.07	75.24	82.67	75.87
GNDA(0.4) + DCNN + Self-Attention	83.01	79.57	84.02	81.89	87.37	85.22
GNDA(0.2, 0.4) + DCNN + Self-Attention	84.77	81.78	86.99	83.05	88.55	84.69
GNDA(0.2, 0.4, 0.6) + DCNN + Self-Attention	86.34	81.87	88.85	84.41	90.26	86.51

**Table A2:** Final performance metrics of all models with and without Gaussian noise data augmentation based on the SWS strategy (ΔS is fixed at 30 s) with SF=5 Hz, 10 Hz, and 50 Hz and T=300 s by 7-fold cross-validation for sleep stage classification on the SDCP dataset (Macro F1-Score = MF1, Accuracy = ACC, Gaussian Noise Data Augmentation = GNDA, GNDA is applied only to the training folds during cross-validation, highest performances are highlighted in bold).

Model	5 Hz		10 Hz		50 Hz	
	ACC	MF1	ACC	MF1	ACC	MF1
DWT + SVM without GNDA	50.87	43.39	55.21	50.09	67.01	53.79
GNDA(0.4) + DWT + SVM	53.08	44.24	55.57	50.06	68.92	52.97
GNDA(0.2, 0.4) + DWT + SVM	56.60	50.01	59.89	50.85	69.92	52.99
GNDA(0.2, 0.4, 0.6) + DWT + SVM	61.02	51.59	62.89	53.00	69.94	54.18
DCNN without GNDA	73.54	63.08	75.26	64.27	77.90	66.11
GNDA(0.4) + DCNN	75.88	64.22	79.21	66.19	80.67	71.39
GNDA(0.2, 0.4) + DCNN	82.69	71.57	85.08	71.59	86.02	75.88
GNDA(0.2, 0.4, 0.6) + DCNN	83.34	72.01	84.61	72.03	85.81	76.11
RNN-based attention without GNDA	66.74	60.20	66.92	61.57	71.03	64.51
GNDA(0.4) + RNN-based attention	67.45	61.24	68.48	61.03	70.44	63.06
GNDA(0.2, 0.4) + RNN-based attention	66.24	60.07	67.51	61.11	68.76	62.28
GNDA(0.2, 0.4, 0.6) + RNN-based attention	68.30	62.31	69.87	64.22	70.56	66.47
Self-attention without GNDA	75.01	65.23	75.89	66.04	77.29	68.27
GNDA(0.4) + Self-attention	78.87	70.34	80.33	72.48	80.67	73.88
GNDA(0.2, 0.4) + Self-attention	75.21	68.55	78.99	69.30	80.05	72.97
GNDA(0.2, 0.4, 0.6) + Self-attention	78.59	70.40	80.64	73.01	81.15	74.19
GNDA(0.4) + DCNN + Self-Attention	82.08	78.24	85.19	80.03	86.24	81.89
GNDA(0.2, 0.4) + DCNN + Self-Attention	85.57	81.78	87.69	83.56	88.06	83.18
GNDA(0.2, 0.4, 0.6) + DCNN + Self-Attention	86.38	81.81	88.84	83.08	**88.56**	**83.57**

## Appendix C. Sensor Channel Test on the SDCP Dataset

This appendix provides the details of the sensor channel test using our proposed GNDA-based DCSAM with SF=50 Hz, T=30 s, and δ=(0.2,0.4,0.6) on the SDCP dataset. Not only all single sensor channels but also combinations between different sensor channels were tested.

We first calculate a *Jacobian Score* (JS) as an evaluation measure to indicate the relevance between a sensor channel and the performance of sleep stage classification. A neural network outputting softmax scores can be treated as a multivariate, vector-valued function $F:R^{T\times C}\to R^{\lambda }$ where *T*, *C*, and λ present the length of the input time window, the dimension of the sensor channel, and the number of sleep stages, respectively. Assuming that $\psi \in R^{T\times C}$ is the model input and associated softmax output *y* can be defined as $F_{\theta }\left(\psi \right)$ = ($y_{1},\cdots ,y_{\lambda }$) $\in R^{\lambda }$ where θ is the set of parameters of the model. A Jacobian value $J_{F_{\theta }\left(\psi \right)}$ can be formed as follows:

(A1) $$J_{F_{\theta }\left(\psi \right)}=\frac{\partial y_{i}}{\partial \psi_{t,c}},$$

$J_{F_{\theta }\left(\psi \right)}$ represents the partial derivatives of each element of the output *y* with respect to each element of the input ψ. As known, derivatives indicate how modifying each element of ψ would impact each element of *y* (i.e., prediction for each class). $J_{F_{\theta }\left(\psi \right)}$ with a large absolute value means that the corresponding input significantly matters classification results. Then, the JS $\Omega_{c}$ for channel *c* is computed by averaging absolute $J_{F_{\theta }\left(\psi \right)}$s over all *T* and λ:

(A2) $$\Omega_{c}=\frac{1}{\lambda |T|}\sum_{i=1}^{\lambda }\sum_{t=1}^{T}\left|J_{F_{\theta }\left(\psi \right)}\right|,$$

where t∈[1,T], c∈[1,C], and i∈[1,λ]. $\Omega_{c}$ represents the overall importance of the *c*th channel for the sleep stage classification of input ψ. The final $\Omega_{c}$ for each sensor channel is calculated based on 7-fold cross-validation and then averaged over all folds. As shown in Table A3, all EEG channels were able to obtain relatively high scores, implying their important contributions to our sleep stage classification task.

**Table A3:** Comparison of sensor channels’ contributions to sleep stage classification based on their Jacobian scores.

Sensor Modality	Sensor Channel	Jacobian Score ($\Omega_{c}$)
EEG	C4M1	0.1827
EEG	C3M2	0.1752
EOG	LEOGM2	0.1748
EOG	REOGM1	0.1600
EEG	O2M1	0.1442
EEG	F4M1	0.1299
EEG	F3M2	0.1225
EEG	O1M2	0.1060
EMG	Chin EMG	0.0244
EMG	Leg (left)	0.0109
EMG	Leg (right)	0.0087

Second, we also performed further channel tests to emphasize the importance of multiple sensor channels. Different sensor modalities and sensor channels were investigated based on the 7-fold cross-validation using GNDA-based DCSAM with T=30 s, SF=50 Hz and δ=(0.2,0.4,0.6), which is the configuration yielding the best performance on the SDCP dataset. The results of channel testing are presented in Table A4. We attempt to test single-channel EEG, single-channel EOG, single-channel EMG, and various combinations of EEG, EOG, and EMG channels. In general, sleep analysis uses corresponding pairs of detected EEGs to classify sleep stages, e.g., *C3M2* is combined with *C4M1*, but in our study, we test more diverse combinations of sensor modalities and channels.

Table A4 shows that using 6 EEG channels is more efficient than using 2 EEG channels and single-channel EEG. Moreover, using 2 EOG channels can also achieve strong performance. However, the performance is still lower than that obtained with both EEG and EOG modalities (6 EEG channels and 2 EOG channels). The EOG modality brings performance improvement, which demonstrates that our GNDA-based DCSAM exploits more correlative features of each sleep stage from 2 EOG channels in the long sleep monitoring context. The use of three EMG channels results in poor performance, but combining them with 6 EEG and 2 EOG channels, namely using all the 11 sensor channels leads to the best performance.

**Table A4:** Influence of sensor channels for our GNDA-based DCSAM with T=30 s, SF=50 Hz and δ=(0.2,0.4,0.6) on the SDCP dataset (Macro F1-Score = MF1, Accuracy = ACC), highest performance is highlighted in red.

Sensor Channel	T=30 s, SF=50 Hz		Sensor Channel	T=30 s, SF=50 Hz	
	ACC	MF1		ACC	MF1
C3M2	82.89	76.31	C3M2 + O2M1	80.19	72.07
C4M1	83.77	78.09	C4M1 + O1M2	85.28	80.20
F3M2	79.09	72.24	F3M2 + O2M1	83.73	78.91
F4M1	78.52	73.19	F4M1 + O1M2	80.54	74.85
O1M2	69.18	63.66	6 EEG channels	87.64	82.19
O2M1	72.43	64.04	REOGM1 (EOG)	83.95	78.12
C3M2 + C4M1	83.58	78.10	LEOGM2 (EOG)	84.02	76.53
F3M2 + F4M1	81.50	77.00	2 EOG channels	84.98	79.50
O1M2 + O2M1	68.22	61.28	6 EEG + 2 EOG	88.14	83.64
C3M2 + F4M1	84.69	77.59	3 EMG channels	42.21	32.34
C4M1 + F3M2	82.51	75.80	All 11 sensor channels	90.26	86.51
